# Sirt1 overexpression improves senescence‐associated pulmonary fibrosis induced by vitamin D deficiency through downregulating *IL‐11* transcription

**DOI:** 10.1111/acel.13680

**Published:** 2022-07-30

**Authors:** Jiawen Zhou, Haiyun Chen, Qiuyi Wang, Sihan Chen, Rong Wang, Ziyang Wang, Cuicui Yang, Ao Chen, Jingyu Zhao, Zihao Zhou, Zhiyuan Mao, Guoping Zuo, Dengshun Miao, Jianliang Jin

**Affiliations:** ^1^ Department of Human Anatomy, Research Centre for Bone and Stem Cells, Key Laboratory for Aging & Disease The State Key Laboratory of Reproductive Medicine, Nanjing Medical University Nanjing China; ^2^ Anti‐Aging Research Laboratory, Friendship Plastic Surgery Hospital Nanjing Medical University Nanjing China; ^3^ The Laboratory Centre for Basic Medical Sciences Nanjing Medical University Nanjing China

**Keywords:** cell senescence, IL‐11, pulmonary fibroblasts, pulmonary fibrosis, senescence‐associated secretory phenotype, Sirt1, Smad2, vitamin D

## Abstract

Determining the mechanism of senescence‐associated pulmonary fibrosis is crucial for designing more effective treatments for chronic lung diseases. This study aimed to determine the following: whether Sirt1 and serum vitamin D decreased with physiological aging, promoting senescence‐associated pulmonary fibrosis by activating TGF‐β1/IL‐11/MEK/ERK signaling, whether Sirt1 overexpression prevented TGF‐β1/IL‐11/MEK/ERK signaling‐mediated senescence‐associated pulmonary fibrosis in vitamin D‐deficient (*Cyp27b1* ^*−/−*^) mice, and whether Sirt1 downregulated *IL‐11* expression transcribed by TGF‐β1/Smad2 signaling through deacetylating histone at the *IL‐11* promoter in pulmonary fibroblasts. Bioinformatics analysis with RNA sequencing data from pulmonary fibroblasts of physiologically aged mice was conducted for correlation analysis. Lungs from young and physiologically aged wild‐type (WT) mice were examined for cell senescence, fibrosis markers, and TGF‐β1/IL‐11/MEK/ERK signaling proteins, and 1,25(OH)_2_D_3_ and IL‐11 levels were detected in serum. Nine‐week‐old WT, *Sirt1* mesenchymal transgene (*Sirt1* ^*Tg*^), *Cyp27b1* ^*−/−*^, and *Sirt1* ^*Tg*^ *Cyp27b1* ^*−/−*^ mice were observed the pulmonary function, aging, and senescence‐associated secretory phenotype and TGF‐β1/IL‐11/MEK/ERK signaling. We found that pulmonary Sirt1 and serum vitamin D decreased with physiological aging, activating TGF‐β1/IL‐11/MEK/ERK signaling, and promoting senescence‐associated pulmonary fibrosis. Sirt1 overexpression improved pulmonary dysfunction, aging, DNA damage, senescence‐associated secretory phenotype, and fibrosis through downregulating TGF‐β1/IL‐11/MEK/ERK signaling in *Cyp27b1* ^*−/−*^ mice. Sirt1 negatively regulated *IL‐11* expression through deacetylating H3K9/14ac mainly at the region from −871 to −724 of *IL‐11* promoter, also the major binding region of Smad2 which regulated *IL‐11* expression at the transcriptional level, and subsequently inhibiting TGF‐β1/IL‐11/MEK/ERK signaling in pulmonary fibroblasts. This signaling in aging fibroblasts could be a therapeutic target for preventing senescence‐associated pulmonary fibrosis induced by vitamin D deficiency.

## Graphical abstract

Pulmonary Sirt1 and serum vitamin D (VD) decrease with physiological aging, activating TGF‐β1/IL‐11/MEK/ERK (TIME) signaling and promoting senescence‐associated pulmonary fibrosis (SAPF). Sirt1 overexpression ameliorates SAPF in VD‐deficient mice through downregulating *IL‐11* transcribed by Smad2 via deacetylating H3K9/14ac mainly at the region from −871 to −724 of *IL‐11* promoter, and subsequently inhibiting TIME signaling in pulmonary fibroblasts. This signal in aging fibroblasts could be a therapeutic target for preventing physiologically VD deficiency‐induced SAPF.

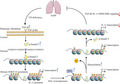

**Abbreviations**
CCK8: Cell Counting Kit‐8
ChIP: chromatin immunoprecipitation
COPD: chronic obstructive pulmonary disease
IL‐11Rα1: interleukin 11 receptor alpha chain 1
IPF: idiopathic pulmonary fibrosis
Masson: Masson's trichrome
PF: pulmonary fibrosis
RII: receptor‐type 2
SAPF: senescence‐associated pulmonary fibrosis
SASP: senescence‐associated secretory phenotype
SA‐β‐gal: senescence‐associated β‐galactosidase
SFTPC: surfactant protein C
SMA: smooth muscle actin
TE: peak expiratory flow relative to total expiratory time
Tg: transgene
TIME: TGF‐β1/IL‐11/MEK/ERK
VD: vitamin D
VDR: VD receptor
WT: wild type

## 1 INTRODUCTION

Chronic lung disease carries a huge health burden worldwide, particularly in older people (Ekezie et al., 2021). The causes of chronic lung diseases, particularly chronic obstructive pulmonary disease (COPD) and idiopathic pulmonary fibrosis (IPF), are now mainly considered to be driven by cellular senescence (Barnes et al., 2019). Determining the mechanism of senescence‐associated pulmonary fibrosis (SAPF) is crucial for designing more effective treatments.

Vitamin D (VD) deficiency is an important feature of physiological aging, and its concentration tends to decrease with aging (Berridge, 2016; Chapuy et al., 1992). What is remarkable about all of these cellular aging processes is that their activity is regulated by VD (Berridge, 2017). It is traditionally believed that VD supplementation prevents the senescence of bone and muscle cells and maintains or even improves their health (Chen, Hu, et al., 2020; Domingues‐Faria et al., 2014; Zhang et al., 2019). More evidence demonstrates that VD deficiency exacerbates aging of various cells (Berridge, 2017; Chen, Hu, et al., 2020; Chen et al., 2019; Domingues‐Faria et al., 2014; Yang et al., 2022). VD deficiency is also associated with pulmonary diseases. Asthma and COPD patients usually suffer from VD deficiency, and low 25‐hydroxyvitamin D levels may represent a cause or a consequence of these conditions (Jolliffe et al., 2020). VD deficiency aggravates bleomycin‐induced pulmonary fibrosis through activating TGF‐β/Smad2 signaling (Li et al., 2019). A clinical report shows that VD deficiency contributes to acute respiratory distress syndrome caused by SARS‐CoV‐2 and that case–fatality rates increase with age (Vyas et al., 2021). VD deficiency is associated with inflammatory reactions and immune dysfunction, which leads to susceptibility to COVID‐19 (Cutolo et al., 2020). However, the exact link between pulmonary fibrosis, VD deficiency, and their relation to cellular senescence is still unclear. Whether VD deficiency could cause SAPF by inducing senescence and profibrotic senescence‐associated secretory phenotype (SASP) of fibroblasts needs to be investigated.

The expression of Sirt1 diminishes with physiological aging in mice (Chen, Zhou, et al., 2020). The mammalian Sir2 ortholog Sirt1 plays an important role in metabolic regulation (Satoh et al., 2013). A loss of Sirt1 may participate in the pathogenesis of pulmonary fibrosis (PF). Thus, its activation could be an effective treatment for the early and late stages of PF (Chu et al., 2018; Liu et al., 2022). Sirt1 is predominantly a histone deacetylase that directly modifies nucleosome histones, thereby regulating chromatin structure and gene expression (Mazumder et al., 2020). Sirt1‐mediated histone deacetylation is associated with transcriptional repression. However, whether Sirt1 prevents VD‐deficiency‐induced SAPF through modifying the promoter of profibrotic molecular transcribed by TGF‐β/Smad2 signaling is still unknown.

We previously found that IL‐11 mediated noncanonical TGF‐β1/IL‐11/MEK/ERK (TIME) signaling to induce SAPF by promoting cell senescence as well as stimulating TGF‐β1 and IL‐11 secretion and collagen 1 synthesis in aging pulmonary fibroblasts (Chen, Chen, et al., 2020). Fibroblast‐specific IL‐11 signaling directly contributes to PF (Ng et al., 2020). However, whether TIME signaling mediates SAPF in mice with VD deficiency is unknown. TGF‐β1 exerts its biological effects by activating downstream transcription factors including Smad2 and Smad3 (Hu et al., 2018). Previous studies have shown that Smad2 acts as a core transcription factor in the TGF‐β1 signaling pathway during multiple organ fibrosis (Hu et al., 2018; Koo et al., 2016). TGF‐β1/Smad2 signaling promotes upregulation of *IL‐11* mRNA in cardiac fibroblasts (Schafer et al., 2017). However, whether TGF‐β1/Smad2 transcribes *IL‐11* in pulmonary fibroblasts is still unclear, and whether Sirt1 downregulates *IL‐11* expression through histone deacetylation at the promoter of Smad2 in pulmonary fibroblasts is also unknown.

The present study demonstrated that pulmonary Sirt1 and serum VD decreased with physiological aging, activating TIME signaling, and promoting SAPF. Sirt1 overexpression ameliorated SAPF in mice with VD deficiency, through downregulating *IL‐11* transcribed by Smad2 via deacetylating H3K9/14ac mainly at the region from −871 to −724 of *IL‐11* promoter, and subsequently inhibiting TIME signaling in pulmonary fibroblasts. This signaling in aging fibroblasts could be a therapeutic target for preventing VD‐deficiency‐induced SAPF. Sirt1 agonist SRT1720, anti‐IL‐11 neutralizing antibodies, and interleukin 11 receptor alpha chain 1 (IL‐11Rα1) inhibitor could be used for translational therapy of SAPF.

## 2 RESULTS

### 2.1 Pulmonary Sirt1 and serum VD decrease with physiological aging, activating TIME signaling, and promoting SAPF

**FIGURE 1:**
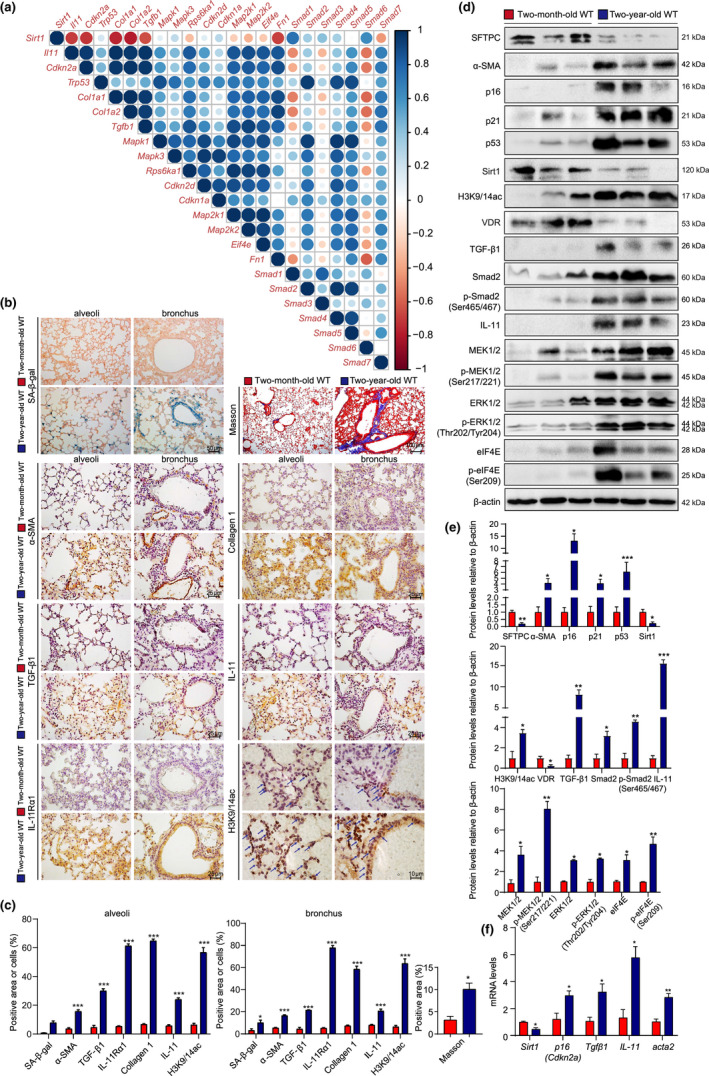
Pulmonary Sirt1 and serum VD decreases with physiological aging, activating TIME signaling, and promoting SAPF. (a) Gene correlation analysis using RNAseq data of pulmonary GFP^+^CD45^−^CD31^−^EpCAM^−^ fibroblasts from physiologically aged (18 months old) Col1α1‐GFP mice treated with or without bleomycin. The lungs from young (2 months old) and physiologically aged (2 years old) WT mice were detected. (b) Representative micrographs of paraffin‐embedded pulmonary tissue stained histochemically for SA‐β‐gal and with Masson's trichrome (Masson), immunohistochemically for α‐SMA, collagen 1, TGF‐β1, IL‐11, IL‐11Rα1, and H3K9/14ac in alveoli and bronchus, with hematoxylin staining the nuclei. (c) Alveolar and bronchial areas positive for Masson staining, α‐SMA, collagen 1, TGF‐β1, IL‐11, IL‐11Rα1, or H3K9/14ac. (d) Western blotting of pulmonary extracts showing SFTPC, α‐SMA, p16, p21, p53, Sirt1, H3K9/14ac, VDR, TGF‐β1, Smad2, p‐Smad2(Ser465/467), IL‐11, MEK1/2, p‐MEK1/2(Ser217/221), ERK1/2, p‐ERK1/2(Thr202/Tyr204), elF4E, and p‐elF4E(Ser209). β‐actin was used as the loading control. (e) Protein levels relative to β‐actin were assessed by densitometric analysis. (f) *Sirt1*, *p16*, *Tgfβ1*, *IL‐11*, and *acta2* mRNA levels in lungs of young and aged mice by real‐time RT‐PCR, calculated as ratio to *β‐actin* mRNA and expressed relative to control. Six mice per group were used for experiments. Values are the mean ± SEM of three determinations per group. *p < 0.05, **p < 0.01, ***p < 0.001 compared with the young mice

To investigate whether SAPF resulted from activation of TIME signaling and decreased Sirt1 level, bioinformatics methods were used to analyze the gene expression profile in aged lungs. After searching the GEO database, the mRNA expression data of pulmonary fibroblasts from physiologically aged (18 months) mice treated with or without bleomycin were analyzed from the GSE191208 dataset. Correlation analysis was conducted and showed that Sirt1 was negatively correlated with cell senescence, SASP, and TIME‐signaling‐related genes (Figure 1a).

To validate these results, the lungs from young (2 months) and physiologically aged (2 years) wild‐type (WT) mice were examined for cell senescence, fibrosis markers, and TIME signaling proteins, and 1,25(OH)_2_D_3_ and IL‐11 levels were detected in serum. There was an increase in senescence‐associated β‐galactosidase (SA‐β‐gal), p16, p53, Masson‐labeled interstitial fibers, α‐smooth muscle actin (SMA), type Ι collagen, TGF‐β1, IL‐11, and IL‐11Rα1 in the lungs of aged compared with young mice. The percentage of H3K9/14ac‐positive cells also increased (Figure 1b,c). However, serum 1,25(OH)_2_D_3_ level, and protein levels of surfactant protein C (SFTPC), VD receptor (VDR) and Sirt1 decreased in lungs of aged compared with young mice (Figure 1b,c; Figure S1D). Cell senescence proteins p16, p21, and p53, and TIME signaling proteins TGF‐β1, Smad2, p‐Smad2(Ser465/467), IL‐11, MEK1/2, p‐MEK1/2(Ser217/221), ERK1/2, p‐ERK1/2(Thr202/Tyr204), elF4E, and p‐elF4E(Ser209), and serum IL‐11 level, as well as H3K9/14ac protein level, significantly increased in lungs of aged compared with young mice (Figure 1d,e; Figure S1E). Real‐time RT‐PCR confirmed downregulated *Sirt1* mRNA level and upregulated *p16*, *Tgfβ1*, *IL‐11*, and *acta2* mRNA levels (Figure 1f).

### 2.2 Sirt1 overexpression improves pulmonary dysfunction in VD‐deficient mice

**FIGURE 2:**
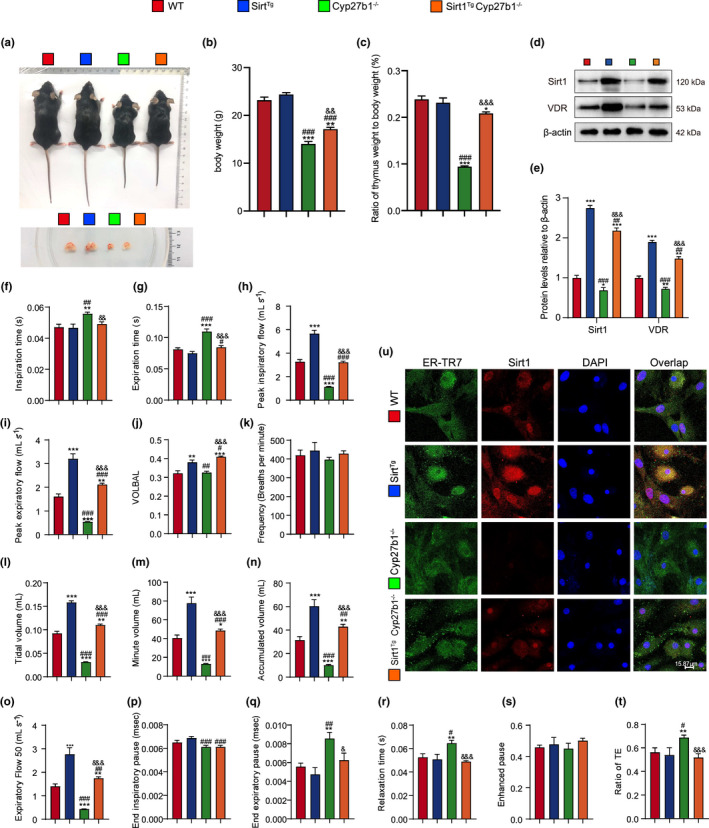
Sirt1 overexpression improves pulmonary dysfunction in VD‐deficient mice. (a) Representative appearances of 9‐week‐old WT, *Sirt1* ^*Tg*^, *Cyp27b1* ^*−/−*^ and *Sirt1* ^*Tg*^ *Cyp27b1* ^*−/−*^ mice and whole view of the thymus. (b, c) Body weight of different genotyped mice and the ratio of thymus weight relative to body weight. (d, e) Western blotting of pulmonary extracts showing Sirt1 and VDR protein levels. Pulmonary function was detected by the whole‐body plethysmography for (f) inspiration time (s), (g) expiration time (s), (h) peak inspiratory flow (mL s^−1^), (i) peak expiratory flow (mL s^−1^), (j) VOLBAL, (k) frequency (breaths per minute), (l) tidal volume (mL), (m) minute volume (mL), (n) accumulated volume (mL), (o) expiratory flow 50 (mL s^−1^), (p) end inspiratory pause (ms), (q) end expiratory pause (ms), (r) relaxation time (s), (s) enhanced pause, and (t) ratio of TE. Six mice per group were used for experiments. (u) Representative micrographs of cells immunofluorescently stained for Sirt1 and ER‐TR7, with DAPI staining the nucleus. Values are the means ± SEM of six determinations per group. *p < 0.05, **p < 0.01, ***p < 0.001 compared with the WT group; ^#^ *p* < 0.05, ^##^ *p* < 0.01, ^###^ *p* < 0.001 compared with the *Sirt1* ^*Tg*^ group; ^&^ *p* < 0.05, ^&&^ *p* < 0.01, ^&&&^ *p* < 0.001 compared with *Cyp27b1* ^*−/−*^ group

As a hallmark of aging, thymic involution manifests through a reduction in thymic mass and size, and hence a decline in thymocytes (Mittelbrunn & Kroemer, 2021). *Cyp27b1* ^*−/−*^ mice showed a significantly smaller body size, body weight, thymus, and ratio of thymus weight relative to body weight, implying an aging phenotype, which was rescued by Sirt1 overexpression (Figure 2a–c). The protein levels of Sirt1 and VDR were all decreased in the lungs of *Cyp27b1* ^*−/−*^ mice when compared with WT mice and then increased in *Sirt1* ^*Tg*^ and *Sirt1* ^*Tg*^ *Cyp27b1* ^*−/−*^ mice (Figure 2d,e). To assess pulmonary function, expiratory and inspiratory‐related indexes were evaluated. Peak inspiratory flow, peak expiratory flow, tidal volume, minute volume, accumulated volume, and expiratory flow 50 decreased significantly, while inspiration time, expiration time, end expiratory pause, relaxation time, and the ratio of peak expiratory flow relative to total expiratory time (TE) increased significantly in *Cyp27b1* ^*−/−*^ mice when compared with WT mice. Pulmonary frequency was not altered among any of the groups. Sirt1 overexpression increased peak inspiratory flow, peak expiratory flow, tidal volume, minute volume, accumulated volume, and expiratory flow 50, and decreased inspiration time, expiration time, end expiratory pause, relaxation time, and ratio of TE (Figure 2f–t).

To confirm whether Sirt1 was overexpressed in pulmonary fibroblasts in *Sirt1* ^*Tg*^ mice, pulmonary fibroblasts were separated from WT, *Sirt1* ^*Tg*^, *Cyp27b1* ^*−/−*^, and *Sirt1* ^*Tg*^ *Cyp27b1* ^*−/−*^ mice. Immunofluorescence of Sirt1 was conducted, showing the increase of Sirt1 in pulmonary fibroblasts in *Sirt1* ^*Tg*^ and *Sirt1* ^*Tg*^ *Cyp27b1* ^*−/−*^ mice when compared with WT mice, while decreased in *Cyp27b1* ^*−/−*^ mice (Figure 2u). These results demonstrated that VD deficiency caused pulmonary dysfunction, which could be ameliorated by Sirt1 overexpression.

### 2.3 Sirt1 overexpression improves pulmonary aging in VD‐deficient mice

To determine whether the rescue of pulmonary dysfunction by Sirt1 overexpression was associated with alterations in cell senescence, the lungs were examined for markers of senescence. There was a significant increase in pulmonary SA‐β‐gal‐, p16‐ and p53‐positive cells in *Cyp27b1* ^*−/−*^ compared with WT mice (Figure S2A–D). Expression of p16, p19, p21, and p53 proteins was increased in *Cyp27b1* ^*−/−*^ compared with WT mice (Figure S2E,F). Sirt1 overexpression decreased pulmonary SA‐β‐gal and p16‐ and p53‐positive cells, as well as expression of p16, p19, p21, and p53.

### 2.4 Sirt1 overexpression improves pulmonary DNA damage and SASP in VD‐deficient mice

To investigate whether pulmonary aging is caused by DNA damage and SASP and ameliorated by Sirt1 overexpression, the lungs were examined for DNA damage and SASP‐related markers. In *Cyp27b1* ^*−/−*^ mice, there were significant increases in 8‐OHdG‐ and CD3e‐positive inflammatory cells and IL‐1β‐, IL‐6‐, and TNF‐α‐positive areas. Sirt1 overexpression decreased the number of 8‐OHdG‐positive cells, CD3e‐positive inflammatory cells, and IL‐1β‐, IL‐6‐, and TNF‐α‐positive areas (Figure S3A–C). Protein levels of phosphorylated CHK2 (p‐CHK2) (Thr68) and phospho‐Histone H2A.X (γ‐H2A.X) (Ser139) were upregulated in the lungs of *Cyp27b1* ^*−/−*^ mice compared with WT mice. SASP‐associated proteins NF‐κB‐p65, p‐p65(Ser536), IκB‐α, p‐IκB‐α(Ser32), IL‐1β, and IL‐6 were also upregulated. Sirt1 overexpression reduced the above DNA damage and SASP‐related protein levels (Figure S3D,E).

### 2.5 Sirt1 overexpression improves PF in VD‐deficient mice

**FIGURE 3:**
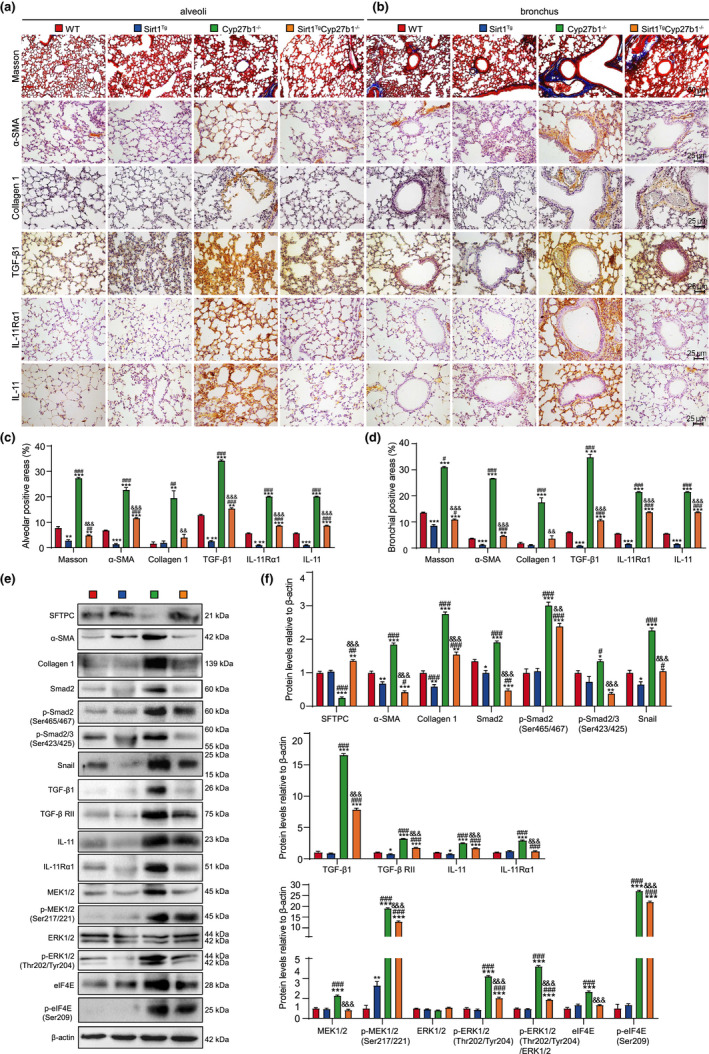
Sirt1 overexpression improves PF through inhibiting TIME signaling in VD‐deficient mice. (a, b) Representative micrographs of paraffin‐embedded pulmonary stained histochemically for Masson's trichrome (Masson), immunohistochemically for α‐SMA, collagen 1, TGF‐β1, IL‐11Rα1, and IL‐11 in alveoli (a) and bronchus (b), with hematoxylin staining the nuclei. Percentage of alveolar (c) and bronchial (d) areas positive for Masson staining, α‐SMA, Collagen 1, TGF‐β1, IL‐11Rα1, or IL‐11. (e) Western blotting of pulmonary extracts showing SFTPC, α‐SMA, Collagen 1, Smad2, p‐Smad2(Ser465/467), p‐Smad2/3(Ser423/425), Snail, TGF‐β1, TGF‐β RII, IL‐11, IL‐11Rα1, MEK1/2, p‐MEK1/2(Ser217/221), ERK1/2, p‐ERK1/2(Thr202/Tyr204), elF4E, and p‐elF4E(Ser209). β‐actin was used as the loading control. (f) Protein expression relative to β‐actin was assessed by densitometric analysis. Six mice per group were used for experiments. Values are the mean ± SEM of six determinations per group. *p < 0.05, **p < 0.01, ***p < 0.001 compared with the WT group; ^#^ *p* < 0.05, ^##^ *p* < 0.01, ^###^ *p* < 0.001 compared with the *Sirt1* ^*Tg*^ group; ^&&^ *p* < 0.01, ^&&&^ *p* < 0.001 compared with the *Cyp27b1* ^*−/−*^ group

To investigate whether VD deficiency led to PF and was ameliorated by Sirt1 overexpression, the lungs were examined for fibrosis markers using immunohistochemistry and Masson trichrome staining. Significant increases were observed in Masson‐labeled interstitial fibers in the lungs of *Cyp27b1* ^*−/−*^ mice. α‐SMA‐, Collagen‐1‐, TGF‐β1‐, IL‐11‐, and IL‐11Rα1‐positive cells or areas were upregulated in the lungs of *Cyp27b1* ^*−/−*^ compared with WT mice. Sirt1 overexpression significantly decreased Masson‐labeled interstitial fibers, as well as the percentage of α‐SMA‐, Collagen‐1‐, TGF‐β1‐, IL‐11‐, and IL‐11Rα1‐positive cells or areas (Figure 3a–d). These results indicated that Sirt1 overexpression ameliorated PF caused by VD deficiency.

### 2.6 Sirt1 overexpression inhibits TIME signaling in VD‐deficient mice

To determine whether PF induced by VD deficiency was mediated by TIME signaling and ameliorated by Sirt1 overexpression, the lungs were examined for TIME‐signaling‐related proteins. In *Cyp27b1* ^*−/−*^ compared with WT mice, there were significant increases in expression of collagen 1 and TGF‐β1/Smad signaling proteins, including mature TGF‐β1, TGF‐β receptor‐type 2 (RII), Smad2, p‐Smad2(Ser465/467), and p‐Smad2/3(Ser423/425). There were significant increases in the expression of IL‐11/MEK/ERK signaling proteins IL‐11, IL‐11Rα1, MEK1/2, p‐MEK1/2(Ser217/221), elF4E, and p‐elF4E(Ser209), and in the ratio of p‐ERK1/2(Thr202/Tyr204) to ERK1/2. However, SFTPC protein was significantly decreased in the lungs of *Cyp27b1* ^*−/−*^ compared with WT mice. Sirt1 overexpression decreased the signaling proteins and upregulated SFTPC protein (Figure 3e,f).

### 2.7 Sirt1 overexpression inhibits cell senescence and TIME signaling, and deacetylates H3K9/14ac in pulmonary fibroblasts from VD‐deficient mice

**FIGURE 4:**
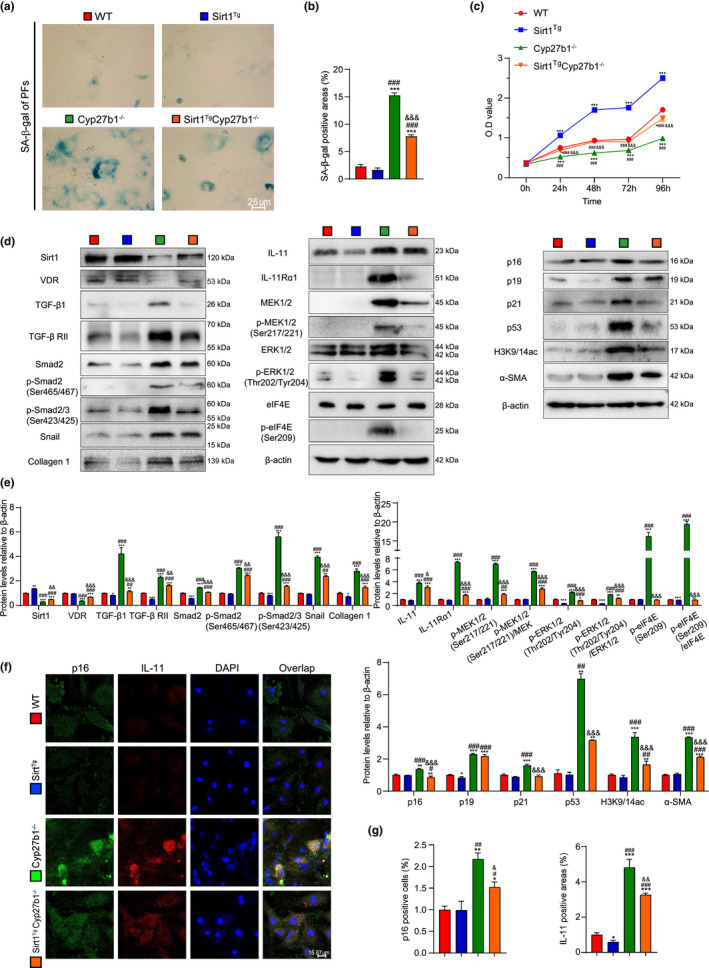
Sirt1 overexpression inhibits cell senescence and TIME signaling, and deacetylates H3K9/14ac in pulmonary fibroblasts from VD‐deficient mice. Pulmonary fibroblasts were isolated from 9‐week‐old WT, *Sirt1* ^*Tg*^, *Cyp27b1* ^*−/−*^ and *Sirt1* ^*Tg*^ *Cyp27b1* ^*−/−*^ mice. (a) Representative micrographs of cells stained cytochemically for SA‐β‐gal. (b) Percentage of SA‐β‐gal‐positive areas relative to the total areas. (c) Second‐passage fibroblast proliferation was determined by CCK‐8 assays and spectrophotometry at 450 nm. Cell number was determined at the indicated times relative to the cell number at hour 0. (d) Western blotting for Sirt1, VDR, TGF‐β1, TGF‐β RII, Smad2, p‐Smad2(Ser465/467), p‐Smad2/3(Ser423/425), Snail, Collagen 1, IL‐11, IL‐11Rα1, MEK1/2, p‐MEK1/2(Ser217/221), ERK1/2, p‐ERK1/2 (Thr202/Tyr204), eIF4E, p‐elF4E(Ser209), p16, p19, p21, p53, acetyl‐histone H3(Lys9/Lys14), and α‐SMA. β‐actin was used as the loading control. (e) Protein expression relative to β‐actin was assessed by densitometric analysis. (f) Representative micrographs of cells immunofluorescently stained for p16 and IL‐11, with DAPI staining the nucleus. (g) The percentage of p16‐positive cells or IL‐11‐positive areas. Three biological replicates were used per experiment. Values are means ± SEM of six determinations. *p < 0.05, **p < 0.01, ***p < 0.001 compared with the WT group; ^#^ *p* < 0.05, ^##^ *p* < 0.01, ^###^ *p* < 0.001 compared with the *Sirt1* ^*Tg*^ group; ^&^ *p* < 0.05, ^&&^ *p* < 0.01, ^&&&^ *p* < 0.001 compared with the *Cyp27b1* ^*−/−*^ group

We investigated whether cell senescence and activation of TIME signaling in pulmonary fibroblasts were responsible for initiating interstitial fibrosis and were ameliorated by Sirt1 overexpression. Pulmonary fibroblasts were separated from 9‐week‐old WT, *Sirt1* ^*Tg*^, *Cyp27b1* ^*−/−*^, and *Sirt1* ^*Tg*^ *Cyp27b1* ^*−/−*^ mice. SA‐β‐gal staining was conducted to detect the senescent cells. Cell Counting Kit‐8 (CCK8) was used to measure cell proliferation. In pulmonary fibroblasts from *Cyp27b1* ^*−/−*^ mice, the percentage of SA‐β‐gal‐positive cells or areas significantly increased compared with those from WT mice. Cell replication slowed in the pulmonary fibroblasts from *Cyp27b1* ^*−/−*^ compared with WT mice (Figure 4a–c).

Proteins related to cell senescence, fibrosis, and TIME signaling were examined. Increases were observed in expression of mature TGF‐β1, TGF‐βRII, Smad2, p‐Smad2(Ser465/467), p‐Smad2/3(Ser423/425), Snail, collagen 1, p16, p19, p21, p53, and α‐SMA and expression of IL‐11 by p16‐labeled‐senescent cells in the pulmonary fibroblasts from *Cyp27b1* ^*−/−*^ compared with WT mice. The IL‐11/MEK/ERK signaling proteins showed significant increases in pulmonary fibroblasts derived from *Cyp27b1* ^*−/−*^ mice. Sirt1 overexpression delayed cell senescence, ameliorated fibrosis, and decreased TIME‐signaling‐related proteins. Sirt1 has been reported as a class III histone deacetylase with important regulatory roles in transcription, cellular differentiation, proliferation, and metabolism (Zerr et al., 2016). We examined the level of H3K9/14ac protein, which was significantly increased in pulmonary fibroblasts from *Cyp27b1* ^*−/−*^ mice, which were then downregulated by Sirt1 overexpression (Figure 4d–g). These results suggested that Sirt1 overexpression ameliorated interstitial fibrosis by inhibiting cell senescence and TIME signaling, and deacetylating H3K9/14ac.

### 2.8 Sirt1 negatively regulates *IL‐11* expression by deacetylating H3K9/14ac in *IL‐11* promoter, mainly the binding region of Smad2

**FIGURE 5:**
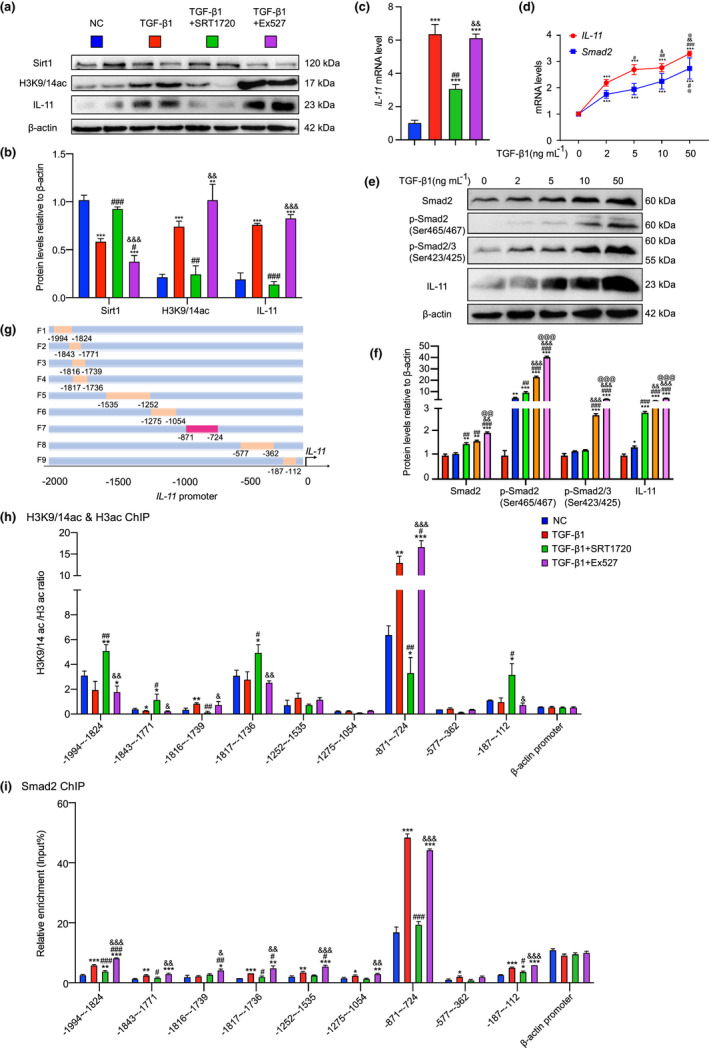
Sirt1 negatively regulates IL‐11 expression through deacetylating H3K9/14ac in *IL‐11* promoter, mainly the binding region of Smad2. MRC‐5 cells were treated by TGF‐β1 (5 ng mL^−1^ 48 h), and SRT1720 (2.5 μM 48 h) or Ex527 (10 μM 48 h). (a) Western blotting of MRC‐5 extracts showing Sirt1, H3K9/14ac, and IL‐11; β‐actin was the loading control. (b) Protein levels relative to β‐actin were assessed by densitometric analysis. (c) *IL‐11* mRNA level in TGF‐β1 induced MRC‐5 cells treated with SRT1720 or Ex527 by real‐time RT‐PCR, calculated as ratio to *β‐actin* mRNA, and expressed relative to control. Three biological replicates were used per experiment. Values are means ± SEM of six determinations. **p < 0.01, ***p < 0.001 compared with control group; ^#^ *p* < 0.05, ^##^ *p* < 0.01, ^###^ *p* < 0.001 compared with TGF‐β1‐treated group; ^&&^ *p* < 0.01, ^&&&^ *p* < 0.001 compared with TGF‐β1‐ and SRT1720‐treated group. (d) *IL‐11* or *Smad2* mRNA level in MRC‐5 cells treated with different concentrations (0, 2, 5, 10 and 50 ng mL^−1^ 48 h) of TGF‐β1. (e) Western blotting of MRC‐5 extracts showing Smad2, p‐Smad2(Ser465/467), p‐Smad2/3(Ser423/425), and IL‐11, β‐actin was the loading control. (f) Protein levels relative to β‐actin were assessed by densitometric analysis. Three biological replicates were used per experiment. Values are means ± SEM of six determinations. *p < 0.05, **p < 0.01, ***p < 0.001 compared with 0 ng mL^−1^ TGF‐β1‐treated group; ^#^p < 0.05, ^##^ *p* < 0.01, ^###^ *p* < 0.001 compared with 2 ng mL^−1^ TGF‐β1‐treated group; ^&^p < 0.05, ^&&^ *p* < 0.01, ^&&&^ *p* < 0.001 compared with 5 ng mL^−1^ TGF‐β1‐treated group; ^@^p < 0.05, ^@@^ *p* < 0.01, ^@@@^ *p* < 0.001 compared with 10 ng mL^−1^ TGF‐β1‐treated group. (g) A model of *IL‐11* promoter truncated primers. (h) ChIP assays were performed with chromatin prepared from MRC‐5 cells treated with TGF‐β1 and SRT1720 or Ex527. The chromatin was immunoprecipitated with normal rabbit IgG or antibodies against H3K9/14ac and H3ac, and precipitated genomic DNA was analyzed through real‐time PCR using different primers for the different regions of *IL‐11* promoter. The β‐actin promoter (−204 to −59 bp) was used as a negative control. (i) ChIP assays were performed with chromatin prepared from MRC‐5 cells treated with TGF‐β1 and SRT1720 or Ex527. The chromatin was immunoprecipitated with normal rabbit IgG or antibodies against Smad2, and precipitated genomic DNA was analyzed through real‐time PCR using different primers for the different regions of *IL‐11* promoter. The *β‐actin* promoter (−204 to −59 bp) was used as a negative control. Three biological replicates were used per experiment. Values are means ± SEM of six determinations. *p < 0.05, **p < 0.01, ***p < 0.001 compared with control group; ^#^ *p* < 0.05, ^##^ *p* < 0.01, ^###^ *p* < 0.001 compared with TGF‐β1‐treated group; ^&^ *p* < 0.05, ^&&^ *p* < 0.01, ^&&&^ *p* < 0.001 compared with TGF‐β1‐ and SRT1720‐ treated group

To investigate the role of Sirt1 in the regulation of PF, we treated MRC‐5 cells with Sirt1 agonist (SRT1720) or inhibitor (Ex527) after inducing fibrosis using TGF‐β1 and analyzed IL‐11 expression. IL‐11 and H3K9/14ac protein levels and *IL‐11* mRNA level were upregulated after being induced with TGF‐β1, while Sirt1 protein level decreased. SRT1720 decreased IL‐11 and H3K9/14ac protein levels and *IL‐11* mRNA level, but increased Sirt1 protein level. Ex527 acted in the opposite way (Figure 5a–c). We treated MRC‐5 cells with or without TGF‐β1 (2, 5, 10, and 50 ng mL^−1^). The increase in TGF‐β1 concentration upregulated mRNA and protein levels of Smad2 and IL‐11, and increased protein expression of p‐Smad2(Ser465/467) and p‐Smad2/3(Ser423/425) (Figure 5d–f). Pulmonary fibroblasts from 9‐week‐old WT mice were isolated and treated by TGF‐β1 and SRT1720 or Ex527. Compared with TGF‐β1 treatment, SRT1720 increased Sirt1 protein level, and decreased H3K9/14ac, Smad2, p‐Smad2(Ser465/467), p‐Smad2/3(Ser423/425), and IL‐11 protein levels. However, Ex527 treatment decreased Sirt1 protein level, and increased H3K9/14ac, p‐Smad2(Ser465/467), p‐Smad2/3(Ser423/425), and IL‐11 protein levels (Figure S4A,B).

To explore whether Sirt1 affected the binding of acetylated histones on the *IL‐11* promoter, the level of histone targets Sirt1 and H3K9/14ac was assayed on 1882 bp of the *IL‐11* promoter from −1994 to −112 in MRC‐5 cells induced with TGF‐β1 and treated with SRT1720 or Ex527. A series of human *IL‐11* promoter primers harboring different polymorphisms were constructed (Figure 5g). H3K9/14ac level was significantly higher in the region from −871 to −724 of the *IL‐11* promoter than in other regions. TGF‐β1 significantly upregulated H3K9/14ac level in the region from −871 to −724 of *IL‐11* promoter compared with the control group, but was downregulated after treatment with SRT1720 and further upregulated after treatment with Ex527 (Figure 5h). IL‐11 protein and mRNA levels changed with Smad2; therefore, we supposed that Smad2 might bind to the *IL‐11* promoter and regulate its expression. The chromatin immunoprecipitation (ChIP) results verified our hypothesis. Smad2 level was significantly higher in the region from −871 to −724 of *IL‐11* promoter than in other regions, increasing significantly after induction with TGF‐β1, downregulated after treatment with SRT1720 and upregulated after treatment with Ex527 (Figure 5i; Figure S4C). These results suggested that Sirt1 negatively regulated *IL‐11* expression through deacetylating H3K9/14ac in the *IL‐11* promoter, mainly the binding region of Smad2.

### 2.9 Smad2 regulates *IL‐11* expression at the transcriptional level

**FIGURE 6:**
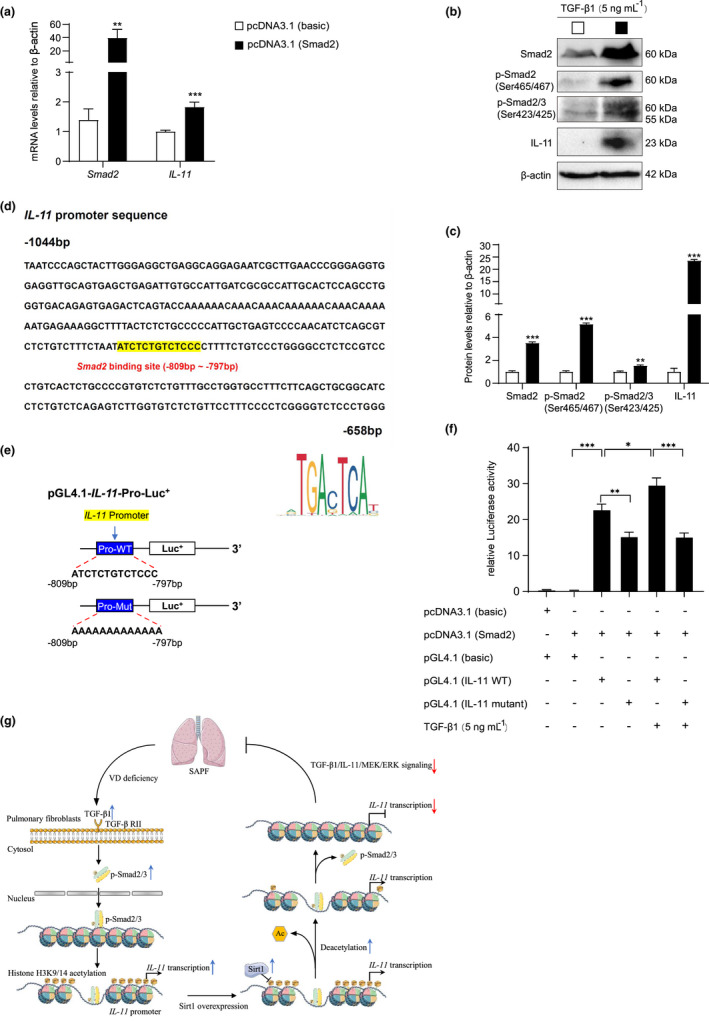
Smad2 regulates *IL‐11* expression at the transcriptional level. MRC‐5 cells were transfected with Smad2 and vehicle (basic) overexpression plasmid. (a) *Smad2* and *IL‐11* mRNA levels in MRC‐5 cells by real‐time RT‐PCR, calculated as ratio to *β‐actin* mRNA and expressed relative to control. (b) Western blotting of MRC‐5 extracts showing Smad2, p‐Smad2(Ser465/467), p‐Smad2/3(Ser423/425), and IL‐11; β‐actin was the loading control. (c) Protein levels relative to β‐actin were assessed by densitometric analysis. Values are means ± SEM of six determinations. **p < 0.01, ***p < 0.001 compared with control group. (d) *Smad2*‐like sequence binding site (highlighted with yellow) in *IL‐11* promoter region and the Smad2 sequence highlighted in red color. (e) Structure schematic diagram of *pGL4.1‐IL‐11* promoter reporter plasmid and mutant *pGL4.1‐IL‐11* promoter reporter plasmid. (f) *IL‐11* promoter activity was measured by a luciferase reporter gene assay. Six biological replicates were used per experiment. Values are means ± SEM of six determinations. *p < 0.05, **p < 0.01, ***p < 0.001 compared with the corresponding group. (g) TGF‐β1 level increased in the progress of SAPF caused by VD deficiency, leading to activation of Smad2 and formation of p‐Smad2/3(Ser423/425) complex, which then bound to the promoter region and increased acetylation levels of H3K9/14. Ultimately, *IL‐11* transcription level was upregulated, activating MEK/ERK signaling, which finally caused SAPF. Sirt1 overexpression ameliorated SAPF in VD‐deficient mice through downregulating *IL‐11* transcribed by Smad2 via deacetylating H3K9/14ac, and subsequently inhibiting TIME signals in pulmonary fibroblasts

To confirm whether Smad2 regulated *IL‐11* expression at the transcriptional level, a Smad2 overexpression plasmid was constructed and transfected into MRC‐5 cells. Smad2 mRNA and protein levels were detected to verify overexpression. IL‐11 mRNA and protein levels were increased in the Smad2‐overexpressed group compared with the control group (Figure 6a–c). We identified a *Smad2*‐like sequence at 2000 bp upstream of the *IL‐11* gene (JASPAR CORE database; http://jaspar.genereg.net/; Figure 6d). Therefore, human *IL‐11* promoter luciferase reporter plasmids with or without *Smad2*‐like sequence (−809 to −797) mutant were constructed. The luciferase activity assay demonstrated that luciferase expression levels were increased significantly in MRC‐5 cells transfected with a Smad2‐binding sequencing plasmid compared with the vehicle, and luciferase expression was increased by TGF‐β1. In contrast, luciferase activity decreased in MRC‐5 cells transfected with a pGL4.1‐*IL‐11* mutant plasmid compared with a Smad2 binding sequencing plasmid. TGF‐β1 could not further activate the mutant reporter (Figure 6e,f). Therefore, Smad2 regulates *IL‐11* expression at the transcriptional level.

## 3 DISCUSSION

In this study, pulmonary Sirt1 and serum VD decreased with physiological aging, activating TIME signaling, and promoting SAPF. VD deficiency led to pulmonary dysfunction, DNA damage, SASP, and PF, which could be improved by Sirt1 overexpression. Sirt1 inhibited the TIME signaling activated by the absence of VD. We also observed in pulmonary fibroblasts from VD‐deficient mice that Sirt1 overexpression negatively regulated cell senescence and TIME signaling, and deacetylated H3K9/14ac. Fibroblast‐specific IL‐11, which directly contributed to PF, was reduced by overexpression of Sirt1. ChIP assays showed that Sirt1 inhibited *IL‐11* transcribed by Smad2 through deacetylating H3K9/14ac, mainly at the region from −871 to −724 of *IL‐11* promoter. It was verified by Luciferase reporter gene assay that *IL‐11* expression was regulated by Smad2 at the transcriptional level. This study not only identified the function of VD deficiency in SAPF, but also proved that TGF‐β1/Smad2 regulated *IL‐11* transcriptionally. Sirt1 downregulated *IL‐11* to ameliorate SAPF induced by VD deficiency through deacetylating H3K9/14ac, and subsequently inhibiting TIME signaling in pulmonary fibroblasts.

It has been argued that VD can precisely regulate cell aging by regulating the various cellular processes that control aging (Berridge, 2017). It is reported that 1,25(OH)_2_D_3_ has antiaging effects via upregulating nuclear respiratory factor (Nrf)2, reducing ROS, decreasing DNA damage, inhibiting p16/Rb and p53/p21 signaling, increasing cell proliferation, and reducing cellular senescence and SASP (Chen et al., 2019). However, the role of VD in pulmonary function and lung pathology is seldom reported. There is evidence that *Cyp27b1* gene knockout, leading to VD deficiency, exacerbates bleomycin‐induced PF through activating TGF‐β/Smad2/3 signaling (Li et al., 2019). A different opinion holds that VD induces cellular senescence and aggravates the lung pathology during the presence of DNA damage induced by bleomycin (Guijarro et al., 2018). A clinical survey showed that VD level was lower in patients with interstitial lung disease and associated with poor prognosis, and increasing VD levels after treatment indicated an improvement in pulmonary function (Gao et al., 2020). Low VD status is associated with increased susceptibility to infectious disease, notably, upper respiratory tract infections from COVID‐19 (Mitchell, 2020). VD supplementation could be especially important for older people as they are at high risk of poor outcome from COVID‐19 (Cutolo et al., 2020; Schafer et al., 2017). Here, we used a *Cyp27b1* global knockout mouse model, which showed insufficient synthesis of active VD, and found that VD deficiency caused premature aging of mice with decreased size of thymus and degraded lung function. Increased numbers of p16‐, p19‐, p21‐, p53‐, and SA‐β‐gal‐positive cells in pulmonary cells of *Cyp27b1* null mice indicated the senescence of the cells after VD deficiency. Increased levels of inflammation‐related molecules IL‐1β, IL‐6, TNF‐α, p‐p65(Ser536), and p‐IκB‐α(Ser32), and CD3e‐positive T cells showed increased T‐cell invasion and inflammation levels after VD deficiency. DNA damage increased as demonstrated by raised levels of p‐CHK2(Thr68)‐, γ‐H2A.X(Ser139)‐ and 8‐OHdG‐positive cells in the lungs. These results demonstrated that compared with WT littermates, VD‐deficient mice showed increased levels of inflammation, DNA damage, and cell senescence.

Research on tissue fibrosis has recently focused on IL‐11 (Ng et al., 2020; Ren et al., 2019; Schafer et al., 2017). Our previous study has also shown that IL‐11 is highly expressed in the prematurely aged lung tissues of mice (Chen, Chen, et al., 2020). TGF‐β1 increased Smad‐dependent *IL‐11* expression, thus aggravating cardiovascular fibrosis (Schafer et al., 2017). IL‐11 is upregulated in the lungs of patients with IPF and is secreted by IPF fibroblasts (Ng et al., 2019). A recent study found that TGF‐β1/Smad2 signaling promoted upregulation of *IL‐11* mRNA in cardiac fibroblasts (Schafer et al., 2017). However, whether VD deficiency causes PF by increasing Smad2‐dependent *IL‐11* transcription is still unclear. A previous study showed higher Smad2 level in patients with severe compared with mild fibrosis, while Smad3 expression was not much different in the patient groups (Koo et al., 2016). Furthermore, we used the website (https://jaspar.genereg.net/) to predict the binding sites of Smad2 or Smad3 in *IL‐11* promoter, and found that Smad2 had 32 predicted binding sites, while Smad3 only had three. See “Predicting the binding sites of Smad2 or Smad3 in *IL‐11* promoter” in Supporting Information S4. Thus, we chose to investigate Smad2 in this study. Our study demonstrated that IL‐11 protein level was upregulated during VD deficiency in pulmonary tissue and fibroblasts. TGF‐β1 treatment significantly increased *IL‐11* mRNA and protein levels. Mechanistic studies have shown that TGF‐β1 transcriptionally regulates *IL‐11* expression in a Smad2‐dependent manner. Previous study (Ding et al., 2013) showed that VDR/Smad co‐occupied the regulatory region of Col1α1 and VDR showed stronger combinatory activity, thus inhibiting transcription of Col1α1 to inhibit liver fibrosis. However, whether VD deficiency mitigates the competitive binding of VDR and Smad2 to the promoter of *IL‐11*, and thus enhancing Smad2 transcribing *IL‐11* needs further study.

Sirt1 is a member of the class III histone deacetylases with important regulatory roles in a variety of pathophysiological processes (Zeng et al., 2017). Accumulating evidence has shown that Sirt1 plays a protective role in preventing cellular metabolism, cell senescence, and inflammation by regulating several signaling pathways, especially via its ability to deacetylate target proteins (Fang et al., 2021; Xu et al., 2020). Sirt1 not only catalyzes the deacetylation of lysine residues in histone substrates such as H1, H3, and H4, but also removes many acetyl groups from nonhistone substrates (Ren et al., 2019), including peroxisome proliferator‐activated receptor‐γ coactivator‐1α, forkhead box class O family, Nrf1/2, p53, eIF2α (Prola et al., 2017), NF‐κB, and others (Maiese, 2021). Such deacetylation of transcription factors related to metabolism could lead to intracellular changes. It has been reported that Sirt1 attenuates hepatic (Zhao et al., 2018), cardiac (Wu et al., 2021), renal (Liu et al., 2020), and pulmonary (Mazumder et al., 2020; Peterson et al., 1986) fibrosis (Chen, Chen, et al., 2020) when pharmacologically active in mice. Our experiments built upon these studies showed that Sirt1 overexpression ameliorated PF through deacetylating lysine 9/14 of histone H3 mainly at the region from −871 to −724 of *IL‐11* promoter.

Sirt1 activation prevents PF by inhibiting epithelial‐to‐mesenchymal transition (EMT; Rong et al., 2016) and TGF‐β signaling (Wei et al., 2015). TGF‐β1 is the principal profibrotic factor, stimulating the secretion of IL‐11 and finally leading to fibrosis. In therapeutic studies, anti‐IL‐11 treatment diminished lung inflammation and reversed PF, while inhibiting ERK and Smad activation in mice (Ren et al., 2019). In our *in vitro* experiments, MRC‐5 cells were treated with Sirt1 agonist (SRT1720) or inhibitor (Ex527) after inducing fibrosis with TGF‐β1. Consistent with previous studies, TGF‐β1 exposure upregulated IL‐11 level mainly through activating MEK/ERK signaling, leading to senescence and fibrosis. We also found that Sirt1 overexpression inhibited fibrosis signaling by reducing *IL‐11* transcription and phosphorylation of Smad2.

IL‐11 and IL‐11Rα1 are expressed specifically in fibroblasts, in which they drive noncanonical, ERK‐dependent autocrine signaling that is required for fibrogenic protein synthesis (Schafer et al., 2017). Fibroblasts derived from the mesenchyme play a regulatory role in several organs. It is suggested that chronic inflammation occurs because of disordered fibroblast behavior in which failure to switch off their inflammatory program leads to the inappropriate survival and retention of leukocytes within inflamed tissue (Buckley et al., 2001). Our previous study found that IL‐11 was mainly produced by fibroblasts in the progress of SAPF, which is regulated by the TIME signaling pathway. Senescence‐related secretion of TGF‐β1 and IL‐11 and production of collagen 1 by pulmonary fibroblasts stimulated the EMT and senescence of alveolar type II epithelial cells (Chen, Chen, et al., 2020). In our study, VD deficiency caused multiple organ dysfunction. In addition to PF, the thymus of VD‐deficient mice was smaller. Considering that fibroblasts are important sentinel cells in the immune system and play a critical role in the switch from acute inflammation to adaptive immunity and tissue repair (Buckley et al., 2001), we overexpressed *Sirt1* at *Prx1* promoter, which ameliorated PF and improved pulmonary function. Also, senescence and inflammation in other organs have been improved by the extensive role of fibroblasts. Our study focused on the mechanism of Sirt1 overexpression in PF induced by VD deficiency. Further experiments will be conducted to study the role of Sirt1 in other organs for improving dysfunction caused by VD deficiency, such as osteoporosis and muscle atrophy.

A previous population‐based study showed that the prevalence of aging‐related IPF was higher in men (20.2 cases per 100,000) than in women (13.2 cases per 100,000; Zisman et al., 2005). Estrogen decreases with age and pulmonary fibroblasts in IPF exhibit increased responsiveness to estrogen compared with controls, while blockage of estrogen receptor diminishes this effect (Elliot et al., 2019). To exclude the effect of estrogen on SAPF, we only used male mice in this study. Thus, experiments on whether Sirt1 overexpression also ameliorates SAPF in females remain to be conducted.

In summary, our results demonstrated that Sirt1 overexpression ameliorated SAPF in VD‐deficient mice by downregulating *IL‐11* transcribed by Smad2 via deacetylating H3K9/14ac, and subsequently inhibiting TIME signaling in pulmonary fibroblasts. This signaling in aging fibroblasts could be a therapeutic target for preventing VD‐deficiency‐induced SAPF. It is suggested that SAPF could be treated by Sirt1 agonist SRT1720, anti‐IL‐11 neutralizing antibodies, and IL‐11Rα1 inhibitor.

## 4 EXPERIMENTAL PROCEDURES

### 4.1 High‐throughput sequencing analysis

The gene expression profile data were downloaded from Gene Expression Omnibus (https://www.ncbi.nlm.nih.gov/geo/) database. GSE191208 contains the mRNA expression data of GFP^+^CD45^−^CD31^−^EpCAM^−^ pulmonary fibroblasts from physiologically aged (18 months old) Col1α1‐GFP mice treated with or without bleomycin. Before analyzing differentially expressed genes (DEGs) using RStudio (version 4.0.3), unrelated samples were excluded. The correlation between genes was calculated by Pearson correlation analysis.

### 4.2 Mice and genotyping

*Cyp27b1* heterozygous (*Cyp27b1* ^*+/−*^) mice were generated at McGill University (Montreal, Canada) and genotyped by PCR as described previously (Chen et al., 2019). The *Cyp27b1* ^*+/−*^ mice, originally on a BALB/c background, were repeatedly backcrossed with WT mice on a C57BL/6J background for over 12 generations to obtain *Cyp27b1* ^*+/−*^ mice on a C57BL/6J background. *Sirt1* ^*Tg*^ mice that highly express *Sirt1* under the control of a 2.4‐kb *Prx1* promoter were generated in Nanjing Medical University (Nanjing, China), and genotyped by PCR as described previously (Sun, Qiao, et al., 2018). Adult *Cyp27b1* ^*+/−*^ female mice were crossed with *Cyp27b1* ^*+/−*^ male mice (both also maintained on a C57BL/6J genetic background) to obtain *Cyp27b1* ^*−/−*^ male mice. *Sirt1* ^*Tg*^ female mice and *Cyp27b1* ^*+/−*^ male mice were crossed to obtain male mice with the genotypes of WT, *Sirt1* ^*Tg*^, *Cyp27b1* ^*−/−*^, and *Sirt1* ^*Tg*^ *Cyp27b1* ^*−/−*^. All the animals used in this study were male and were weaned at 21 days old. Five male mice per cage were housed separately. All the animals in this study were fed a normal diet, which contained 1.0% calcium, 0.67% phosphorus, and 2.2 IU VD g^−1^ (#1010013; Jiangsu Province Collaborative Medicine Bioengineering Co. Ltd.). This study was performed strictly according to the guidelines of the Institute for Laboratory Animal Research of Nanjing Medical University in Nanjing, China. The protocol was approved by the Committee on the Ethics of Animal Experiments of Nanjing Medical University (Permit Number: IACUC‐1802007).

### 4.3 Cell cultures

#### 4.3.1 Pulmonary fibroblasts

The lungs were separated from 9‐week‐old mice anesthetized and perfused as previously described, minced, and digested to generate pulmonary fibroblasts, which were cultured in 90% α‐MEM (BL306A; Biosharp) with 10% FBS (ZQ500‐S; Zhong Qiao Xin Zhou Biotechnology), 100 U mL^−1^ penicillin, and 0.1 mg mL^−1^ streptomycin (CSP006; Zhong Qiao Xin Zhou Biotechnology).

#### 4.3.2 MRC‐5 cells

Human embryonic lung fibroblasts (MRC‐5 cells) were cultured in 90% MEM (ZQ300; Zhong Qiao Xin Zhou Biotechnology) with 10% FBS (ZQ500‐S; Zhong Qiao Xin Zhou Biotechnology), 100 U mL^−1^ penicillin, 0.1 mg mL^−1^ streptomycin (CSP006; Zhong Qiao Xin Zhou Biotechnology), 1% L‐alanyl‐L‐glutamine (CSP004; Zhong Qiao Xin Zhou Biotechnology), and 1% sodium pyruvate (CSP003; Zhong Qiao Xin Zhou Biotechnology) as previously described (Liu et al., 2021).

### 4.4 Administration of drugs or reagents

#### 4.4.1 Recombinant human TGF‐β1, Sirt1 agonist (SRT1720), and inhibitor (Ex527)

MRC‐5 cells were treated with 2.5 μM SRT1720 (Sun, He, et al., 2018) or 10 μM Ex527 (Shao et al., 2019) for 48 h, and different concentrations (0, 2, 5, 10, and 50 ng mL^−1^) of human TGF‐β1 (Wang et al., 2018).

### 4.5 Cell proliferation

Cell proliferation was analyzed by CCK‐8 assay (#C0038; Beyotime Institute of Biotechnology) as previously described (Jin et al., 2017).

### 4.6 Pulmonary function analysis

Mice were placed in the plethysmography chambers of a whole‐body plethysmograph (WBP‐8MR; TOW‐INT TECH) after 15 min acclimation in the cavity. Over a period of 15 min, unrestrained mice were monitored. The inspiration time, expiration time, peak inspiratory flow, peak expiratory flow, VOLBAL, frequency, tidal volume, minute volume, accumulated volume, expiratory flow 50 (expiratory flow at 50% volume), end expiratory pause, end inspiratory pause, relaxation time, enhanced pause, and ratio of TE (peak expiratory flow relative to total expiratory time) were determined by ResMass version 1.4.2.8 (TOW‐INT TECH) as previously described (Sun et al., 2021; Xiong et al., 2021).

### 4.7 Preparation of pulmonary sections

Pulmonary samples from 9‐week‐old mice anesthetized and perfused as previously described were cut into small pieces, and postfixed in periodate‐lysine‐paraformaldehyde (PLP) solution for 24 h at 4°C as previously described (Chen, Chen, et al., 2020). For histochemistry or immunohistochemistry, sections were dehydrated in a series of graded ethanol solutions, embedded in paraffin, and cut into 5‐μm sections using a rotary microtome (Leica Biosystems Nussloch GmbH) as previously described (Jin et al., 2017).

### 4.8 Histology staining

Serial paraffin sections were deparaffinized and rehydrated for histochemical or immunohistochemical staining.

#### 4.8.1 Pre‐embedding SA‐β‐gal staining

Pulmonary samples from mice were stained as previously described (Jin et al., 2011, 2017).

#### 4.8.2 Masson's trichrome staining

Serial paraffin sections were stained with Masson's detection kit (#D026; Nanjing Jiancheng Bioengineering Institute) as previously described (Jin et al., 2017).

#### 4.8.3 Immunohistochemical staining

Immunohistochemical staining was performed as previously described. Primary antibodies against p16 (ab211542; Abcam), p53 (sc‐126; Santa Cruz Biotechnology), acetyl‐histone H3 (Lys9/Lys14; #9677; Cell Signaling Technology), 8‐OHdG (ab62623; Abcam), IL‐1β (ab9722; Abcam), CD3e (sc‐20,047; Santa Cruz Biotechnology), IL‐6 (sc‐1265; Santa Cruz Biotechnology), TNF‐α (sc‐52,746; Santa Cruz Biotechnology), α‐SMA (ab28052; Abcam), Collagen 1 (#1310‐08; Southern Biotech), TGF‐β1 (ab64715; Abcam), and IL‐11 (sc‐133,063; Santa Cruz Biotechnology), and IL‐11Rα1 (sc‐130,920; Santa Cruz Biotechnology). After washing, the sections were incubated with secondary antibody (biotinylated IgG; Sigma‐Aldrich), washed, and processed using Vectastain ABC‐HRP kits (Vector Laboratories).

#### 4.8.4 Immunofluorescent staining of pulmonary sections

Primary antibodies against ER‐TR7 (sc‐73,355; Santa Cruz Biotechnology), Sirt1 (#9475; Cell Signaling Technology) and Dylight488‐conjugated secondary antibody (goat anti‐mouse IgG, GAM4882; Multi Sciences Biotech, Co. Ltd.), and Dylight594‐conjugated secondary antibody (goat anti‐rabbit IgG, GAR5942; Multi Sciences Biotech) were used.

### 4.9 Cytology staining

Cells seeded on a Lab‐Tek II Chamber Slide™ system (Thermo Fisher Scientific Inc.) were fixed with PLP solution for 1 h as previously described (Chen et al., 2022).

#### 4.9.1 SA‐β‐gal staining

SA‐β‐gal staining was performed by using the senescence β‐Galactosidase staining kit (#C0602; Beyotime Institute of Biotechnology) as previously described (Chen et al., 2022).

#### 4.9.2 Immunofluorescent staining of cells

Primary antibodies against IL‐11 (sc‐133,063; Santa Cruz Biotechnology) and p16 (ab211542; Abcam), Dylight488‐conjugated secondary antibody (goat anti‐mouse IgG, BS10015; Bioworld Technology), and Dylight594‐conjugated secondary antibody (goat anti‐rabbit IgG, GAR5942; Multi Sciences Biotech) were used.

### 4.10 RNA extraction and real‐time RT‐PCR

RNA was extracted from the lungs of 9‐week‐old mice using TRIzol reagent (#15596; Invitrogen). Levels of mRNA in pulmonary samples were quantified by real‐time RT‐PCR as previously described (Chen et al., 2022). See the primers in Table S1.

### 4.11 Western blotting

Western blots were generated as previously described (Chen, Chen, et al., 2020). Primary antibodies against Sirt1 (#9475; Cell Signaling Technology), acetyl‐histone H3 (Lys9/Lys14) (H3K9/14ac) (#9677; Cell Signaling Technology), VDR (ab3508; Abcam), p16 (ab211542; Abcam), p19 (sc‐1665; Santa Cruz Biotechnology), p53 (sc‐126; Santa Cruz Biotechnology), p21 (sc‐471; Santa Cruz Biotechnology), CHK2 (sc‐5278; Santa Cruz Biotechnology), p‐CHK2(Thr68) (#PA5‐104715; Invitrogen), γ‐H2A.X(Ser139) (#80312; Cell Signaling Technology), p‐p65(Ser536) (ab76302; Abcam), NF‐κB‐p65 (#8242; Cell Signaling Technology), IκB‐α (AF1282; Beyotime Biotechnology), p‐IκB‐α(Ser32) (sc‐8404; Santa Cruz Biotechnology), IL‐1β (sc‐52,012; Santa Cruz Biotechnology), IL‐6 (sc‐1265; Santa Cruz Biotechnology), SFTPC (ab211326; Abcam), Collagen 1 (#1310–01; Southern Biotech), α‐SMA (ab28052; Abcam), TGF‐β1 (ab64715; Abcam), TGF‐β RII (sc‐17792, Santa Cruz Biotechnology), Smad2 (sc‐101,153; Santa Cruz Biotechnology), p‐Smad2 (Ser465/467) (#3108; Cell Signaling Technology), p‐Smad2/3 (Ser423/425) (sc‐11,769; Santa Cruz Biotechnology) (ab52903; Abcam), Snail (#3879; Cell Signaling Technology), IL‐11 (sc‐133,063; Santa Cruz Biotechnology), IL‐11Rα1 (sc‐130,920; Santa Cruz Biotechnology), MEK1/2 (sc‐81,504; Santa Cruz Biotechnology), p‐MEK1/2(Ser217/221) (sc‐81,503; Santa Cruz Biotechnology), ERK1/2 (#4695; Cell Signaling Technology), p‐ERK1/2 (Thr202/Tyr204) (#4370; Cell Signaling Technology), eIF4E (sc‐9976; Santa Cruz Biotechnology), p‐eIF4E(Ser209) (#9741; Cell Signaling Technology), and acetyl‐histone H3 (#9733; Cell Signaling Technology) were used. β‐actin (BS6007M; Bioworld Technology) was the loading control for the cytoplasmic fraction and total cell protein.

### 4.12 Elisa

Concentrations of 1,25(OH)_2_D_3_ (YFXEM00860) and IL‐11 (YFXEM00807) in serum from mice were detected using ELISA kits (Yifeixue Biotechnology) as previously described (Chen, Chen, et al., 2020).

### 4.13 ChIP

ChIP was performed by Magna ChIP™ Chromatin Immunoprecipitation A kit (Millipore) using MRC‐5 cells. Antibodies against acetyl‐histone H3 (Lys9/Lys14) (H3K9/14ac) (#9677; Cell Signaling Technology), acetyl‐histone H3 (#06‐599; Millipore), Smad2 (#5339; Cell Signaling Technology), and rabbit IgG (#PP64; Millipore) were used to incubate chromatin samples. The primers for different regions of *IL‐11* promoter used for analyzing the binding sites are listed in Table S1.

### 4.14 Dual luciferase assay

The *Smad2* gene was cloned into the vector pcDNA3.1 (TranSheep Bio Co. Ltd.). The chimeric genes of the *IL‐11* promoter plasmids for transfection experiments were constructed in a pGL4.1‐basic vector (TranSheep Bio Co. Ltd.) by ligating the *luciferase* gene at the 5′‐flanking regions of the gene upstream. MRC‐5 cells were plated into 24‐well culture plates 24 h before transfection. The mixtures of 1 μg each of pcDNA3.1‐basic and pGL4.1‐basic, overexpressed pcDNA3.1 and pGL4.1‐basic, overexpressed pcDNA3.1 and pGL4.1‐mutant (mutating the binding sequence “ATCTCTGTCTCCC” into “AAAAAAAAAAAAA”), overexpressed pcDNA3.1 and pGL4.1‐promoter, overexpressed pcDNA3.1 and pGL4.1‐mutant (with or without TGF‐β1), and overexpressed pcDNA3.1 and pGL4.1‐promoter (with or without TGFβ1) were cotransfected with Firefly luciferase (Fluc)–Renilla luciferase (Rluc) into human MRC‐5 cells with the X‐tremeGENE HP DNA Transfection Reagent (Roche Diagnostics Corp.). Two days later, a commercial kit (Promega Corporation) was used to measure the promoter‐driven luciferase activity.

### 4.15 Statistical analysis

GraphPad Prism version 6.07 software (GraphPad Software Inc.) was used to analyze data as previously described (Chen et al., 2022). Measurement data are described as mean ± SEM fold‐change over the vehicle group and were analyzed using Student's *t*‐test and one‐way ANOVA to compare differences among groups. Qualitative data are described as percentages and were analyzed using chi‐square tests as indicated. *P*‐values were two‐sided, and *p* < 0.05 was considered statistically significant.

## AUTHOR CONTRIBUTIONS

Conceptualization: J.J. and D.M.; Methodology: J.Z., C.H., Q.W., S.C., R.W., Z.W., C.Y., A.C., J.Z., Z.Z., Z.M., G.Z., D.M., and J.J.; Software: J.Z., C.H., Q.W., S.C., R.W., Z.W., and J.J.; Validation: J.Z., C.H., Q.W., S.C., R.W., and J.J.; Data Analysis: J.Z., C.H., Q.W., S.C., R.W., Z.W., C.Y., A.C., and J.J.; Writing–Original Draft: J.Z., C.H., Q.W., and J.J., with help from the other authors; Writing–Review & Editing: R.W. and D.M., with help from the other authors; Project Administration and Supervision: R.W., J.J., and D.M.; Funding Acquisition: J.J. and D.M.

## CONFLICT OF INTEREST

The authors declare no competing interests.

## Supporting information


Appendix S1.

## Data Availability

All data and materials used in the analysis are available to any researcher for purposes of reproducing or extending the analysis.

## References

[acel13680-bib-0001] Barnes, P. J. , Baker, J. , & Donnelly, L. E. (2019). Cellular senescence as a mechanism and target in chronic lung diseases. American Journal of Respiratory and Critical Care Medicine, 200(5), 556–564. 10.1164/rccm.201810-1975TR 30860857

[acel13680-bib-0002] Berridge, M. J. (2016). Vitamin D, reactive oxygen species and calcium signalling in ageing and disease. Philosophical Transactions of the Royal Society of London. Series B, Biological Sciences, 371(1700), 20150434. 10.1098/rstb.2015.0434 27377727 PMC4938033

[acel13680-bib-0003] Berridge, M. J. (2017). Vitamin D deficiency accelerates ageing and age‐related diseases: A novel hypothesis. The Journal of Physiology, 595(22), 6825–6836. 10.1113/JP274887 28949008 PMC5685827

[acel13680-bib-0004] Buckley, C. D. , Pilling, D. , Lord, J. M. , Akbar, A. N. , Scheel‐Toellner, D. , & Salmon, M. (2001). Fibroblasts regulate the switch from acute resolving to chronic persistent inflammation. Trends in Immunology, 22(4), 199–204. 10.1016/s1471-4906(01)01863-4 11274925

[acel13680-bib-0005] Chapuy, M. C. , Arlot, M. E. , Duboeuf, F. , Brun, J. , Crouzet, B. , Arnaud, S. , Delmas, P. D. , & Meunier, P. J. (1992). Vitamin D3 and calcium to prevent hip fractures in elderly women. The New England Journal of Medicine, 327(23), 1637–1642. 10.1056/NEJM199212033272305 1331788

[acel13680-bib-0006] Chen, C. , Zhou, M. , Ge, Y. , & Wang, X. (2020). SIRT1 and aging related signaling pathways. Mechanisms of Ageing and Development, 187, 111215. 10.1016/j.mad.2020.111215 32084459

[acel13680-bib-0007] Chen, H. , Chen, H. , Liang, J. , Gu, X. , Zhou, J. , Xie, C. , Lv, X. , Wang, R. , Li, Q. , Mao, Z. , Sun, H. , Zuo, G. , Miao, D. , & Jin, J. (2020). TGF‐beta1/IL‐11/MEK/ERK signaling mediates senescence‐associated pulmonary fibrosis in a stress‐induced premature senescence model of Bmi‐1 deficiency. Experimental & Molecular Medicine, 52(1), 130–151. 10.1038/s12276-019-0371-7 31959867 PMC7000795

[acel13680-bib-0008] Chen, H. , Hu, X. , Yang, R. , Wu, G. , Tan, Q. , Goltzman, D. , & Miao, D. (2020). SIRT1/FOXO3a axis plays an important role in the prevention of mandibular bone loss induced by 1,25(OH)2D deficiency. International Journal of Biological Sciences, 16(14), 2712–2726. 10.7150/ijbs.48169 33110391 PMC7586429

[acel13680-bib-0009] Chen, H. , Zhou, J. , Chen, H. , Liang, J. , Xie, C. , Gu, X. , Wang, R. , Mao, Z. , Zhang, Y. , Li, Q. , Zuo, G. , Miao, D. , & Jin, J. (2022). Bmi‐1‐RING1B prevents GATA4‐dependent senescence‐associated pathological cardiac hypertrophy by promoting autophagic degradation of GATA4. Clinical and Translational Medicine, 12(4), e574. 10.1002/ctm2.574 35390228 PMC8989148

[acel13680-bib-0010] Chen, L. , Yang, R. , Qiao, W. , Zhang, W. , Chen, J. , Mao, L. , Goltzman, D. , & Miao, D. (2019). 1,25‐Dihydroxyvitamin D exerts an antiaging role by activation of Nrf2‐antioxidant signaling and inactivation of p16/p53‐senescence signaling. Aging Cell, 18(3), e12951. 10.1111/acel.12951 30907059 PMC6516172

[acel13680-bib-0011] Chu, H. , Jiang, S. , Liu, Q. , Ma, Y. , Zhu, X. , Liang, M. , Shi, X. , Ding, W. , Zhou, X. , Zou, H. , Qian, F. , Shaul, P. W. , Jin, L. , & Wang, J. (2018). Sirtuin1 protects against systemic sclerosis‐related pulmonary fibrosis by decreasing proinflammatory and profibrotic processes. American Journal of Respiratory Cell and Molecular Biology, 58(1), 28–39. 10.1165/rcmb.2016-0192OC 28800254 PMC5941307

[acel13680-bib-0012] Cutolo, M. , Paolino, S. , & Smith, V. (2020). Evidences for a protective role of vitamin D in COVID‐19. RMD Open, 6(3), e001454. 10.1136/rmdopen-2020-001454 33372031 PMC7771215

[acel13680-bib-0013] Ding, N. , Yu, R. T. , Subramaniam, N. , Sherman, M. H. , Wilson, C. , Rao, R. , Leblanc, M. , Coulter, S. , He, M. , Scott, C. , Lau, S. L. , Atkins, A. R. , Barish, G. D. , Gunton, J. E. , Liddle, C. , Downes, M. , & Evans, R. M. (2013). A vitamin D receptor/SMAD genomic circuit gates hepatic fibrotic response. Cell, 153(3), 601–613. 10.1016/j.cell.2013.03.028 23622244 PMC3673534

[acel13680-bib-0014] Domingues‐Faria, C. , Chanet, A. , Salles, J. , Berry, A. , Giraudet, C. , Patrac, V. , Denis, P. , Bouton, K. , Goncalves‐Mendes, N. , Vasson, M. P. , Boirie, Y. , & Walrand, S. (2014). Vitamin D deficiency down‐regulates notch pathway contributing to skeletal muscle atrophy in old wistar rats. Nutrition & Metabolism, 11(1), 47. 10.1186/1743-7075-11-47 25317198 PMC4195890

[acel13680-bib-0015] Ekezie, W. , Jenkins, A. R. , Hall, I. P. , Evans, C. , Koju, R. , Kurmi, O. P. , & Bolton, C. E. (2021). The burden of chronic respiratory diseases in adults in Nepal: A systematic review. Chronic Respiratory Disease, 18, 1479973121994572. 10.1177/1479973121994572 34227410 PMC8264743

[acel13680-bib-0016] Elliot, S. , Periera‐Simon, S. , Xia, X. , Catanuto, P. , Rubio, G. , Shahzeidi, S. , el Salem, F. , Shapiro, J. , Briegel, K. , Korach, K. S. , & Glassberg, M. K. (2019). MicroRNA let‐7 downregulates ligand‐independent estrogen receptor‐mediated male‐predominant pulmonary fibrosis. American Journal of Respiratory and Critical Care Medicine, 200(10), 1246–1257. 10.1164/rccm.201903-0508OC 31291549 PMC6857483

[acel13680-bib-0017] Fang, Y. , Wang, X. , Yang, D. , Lu, Y. , Wei, G. , Yu, W. , Liu, X. , Zheng, Q. , Ying, J. , & Hua, F. (2021). Relieving cellular energy stress in aging, neurodegenerative, and metabolic diseases, SIRT1 as a therapeutic and promising node. Frontiers in Aging Neuroscience, 13, 738686. 10.3389/fnagi.2021.738686 34616289 PMC8489683

[acel13680-bib-0018] Gao, Y. , Zhao, Q. , Qiu, X. , Zhuang, Y. , Yu, M. , Dai, J. , Cai, H. , & Yan, X. (2020). Vitamin D levels are prognostic factors for connective tissue disease associated interstitial lung disease (CTD‐ILD). Aging, 12(5), 4371–4378. 10.18632/aging.102890 32167486 PMC7093159

[acel13680-bib-0019] Guijarro, T. , Magro‐Lopez, E. , Manso, J. , Garcia‐Martinez, R. , Fernandez‐Acenero, M. J. , Liste, I. , & Zambrano, A. (2018). Detrimental pro‐senescence effects of vitamin D on lung fibrosis. Molecular Medicine, 24(1), 64. 10.1186/s10020-018-0064-z 30567504 PMC6299997

[acel13680-bib-0020] Hu, H. H. , Chen, D. Q. , Wang, Y. N. , Feng, Y. L. , Cao, G. , Vaziri, N. D. , & Zhao, Y. Y. (2018). New insights into TGF‐beta/Smad signaling in tissue fibrosis. Chemico‐Biological Interactions, 292, 76–83. 10.1016/j.cbi.2018.07.008 30017632

[acel13680-bib-0021] Jin, J. , Tao, J. , Gu, X. , Yu, Z. , Wang, R. , Zuo, G. , Li, Q. , Lv, X. , & Miao, D. (2017). P16 (INK4a) deletion ameliorated renal tubulointerstitial injury in a stress‐induced premature senescence model of Bmi‐1 deficiency. Scientific Reports, 7(1), 7502. 10.1038/s41598-017-06868-8 28790310 PMC5548892

[acel13680-bib-0022] Jin, J. , Zhao, Y. , Tan, X. , Guo, C. , Yang, Z. , & Miao, D. (2011). An improved transplantation strategy for mouse mesenchymal stem cells in an acute myocardial infarction model. PLoS One, 6(6), e21005. 10.1371/journal.pone.0021005 21698117 PMC3117862

[acel13680-bib-0023] Jolliffe, D. A. , Stefanidis, C. , Wang, Z. , Kermani, N. Z. , Dimitrov, V. , White, J. H. , McDonough, J. , Janssens, W. , Pfeffer, P. , Griffiths, C. J. , Bush, A. , Guo, Y. , Christenson, S. , Adcock, I. M. , Chung, K. F. , Thummel, K. E. , & Martineau, A. R. (2020). Vitamin D metabolism is dysregulated in asthma and chronic obstructive pulmonary disease. American Journal of Respiratory and Critical Care Medicine, 202(3), 371–382. 10.1164/rccm.201909-1867OC 32186892 PMC7397796

[acel13680-bib-0024] Koo, J. H. , Lee, H. J. , Kim, W. , & Kim, S. G. (2016). Endoplasmic reticulum stress in hepatic stellate cells promotes liver fibrosis via PERK‐mediated degradation of HNRNPA1 and up‐regulation of SMAD2. Gastroenterology, 150(1), 181–193.e188. 10.1053/j.gastro.2015.09.039 26435271

[acel13680-bib-0025] Li, S. R. , Tan, Z. X. , Chen, Y. H. , Hu, B. , Zhang, C. , Wang, H. , Zhao, H. , & Xu, D. X. (2019). Vitamin D deficiency exacerbates bleomycin‐induced pulmonary fibrosis partially through aggravating TGF‐beta/Smad2/3‐mediated epithelial‐mesenchymal transition. Respiratory Research, 20(1), 266. 10.1186/s12931-019-1232-6 31775746 PMC6882226

[acel13680-bib-0026] Liu, H. , Wu, X. , Gan, C. , Wang, L. , Wang, G. , Yue, L. , Liu, Z. , Wei, W. , Su, X. , Zhang, Q. , Tan, Z. , Yao, Y. , Ouyang, L. , Yu, L. , & Ye, T. (2021). A novel multikinase inhibitor SKLB‐YTH‐60 ameliorates inflammation and fibrosis in bleomycin‐induced lung fibrosis mouse models. Cell Proliferation, 54(7), e13081. 10.1111/cpr.13081 34121240 PMC8249783

[acel13680-bib-0027] Liu, L. , Yu, N. , Leng, W. , Lu, Y. , Xia, X. , & Yuan, H. (2022). 6‐gingerol, a functional polyphenol of ginger, reduces pulmonary fibrosis by activating Sirtuin1. Allergologia et Immunopathologia, 50(2), 104–114. 10.15586/aei.v50i2.533 35257553

[acel13680-bib-0028] Liu, T. , Yang, Q. , Zhang, X. , Qin, R. , Shan, W. , Zhang, H. , & Chen, X. (2020). Quercetin alleviates kidney fibrosis by reducing renal tubular epithelial cell senescence through the SIRT1/PINK1/mitophagy axis. Life Sciences, 257, 118116. 10.1016/j.lfs.2020.118116 32702447

[acel13680-bib-0029] Maiese, K. (2021). Targeting the core of neurodegeneration: FoxO, mTOR, and SIRT1. Neural Regeneration Research, 16(3), 448–455. 10.4103/1673-5374.291382 32985464 PMC7996023

[acel13680-bib-0030] Mazumder, S. , Barman, M. , Bandyopadhyay, U. , & Bindu, S. (2020). Sirtuins as endogenous regulators of lung fibrosis: A current perspective. Life Sciences, 258, 118201. 10.1016/j.lfs.2020.118201 32781070

[acel13680-bib-0031] Mitchell, F. (2020). Vitamin‐D and COVID‐19: Do deficient risk a poorer outcome? The Lancet Diabetes and Endocrinology, 8(7), 570. 10.1016/S2213-8587(20)30183-2 32445630 PMC7239633

[acel13680-bib-0032] Mittelbrunn, M. , & Kroemer, G. (2021). Hallmarks of T cell aging. Nature Immunology, 22(6), 687–698. 10.1038/s41590-021-00927-z 33986548

[acel13680-bib-0033] Ng, B. , Dong, J. , D'Agostino, G. , Viswanathan, S. , Widjaja, A. A. , Lim, W. W. , Ko, N. S. J. , Tan, J. , Chothani, S. P. , Huang, B. , Xie, C. , Pua, C. J. , Chacko, A. M. , Guimarães‐Camboa, N. , Evans, S. M. , Byrne, A. J. , Maher, T. M. , Liang, J. , Jiang, D. , … Cook, S. A. (2019). Interleukin‐11 is a therapeutic target in idiopathic pulmonary fibrosis. Science Translational Medicine, 11(511), eaaw1237. 10.1126/scitranslmed.aaw1237 31554736

[acel13680-bib-0034] Ng, B. , Dong, J. , Viswanathan, S. , Widjaja, A. A. , Paleja, B. S. , Adami, E. , Ko, N. S. J. , Wang, M. , Lim, S. , Tan, J. , Chothani, S. P. , Albani, S. , Schafer, S. , & Cook, S. A. (2020). Fibroblast‐specific IL11 signaling drives chronic inflammation in murine fibrotic lung disease. The FASEB Journal, 34(9), 11802–11815. 10.1096/fj.202001045RR 32656894

[acel13680-bib-0035] Peterson, S. W. , Kyriakis, J. M. , & Hausman, R. E. (1986). Changes in insulin binding to developing embryonic chick neural retina cells. Journal of Neurochemistry, 47(3), 851–855. 10.1111/j.1471-4159.1986.tb00689.x 3525755

[acel13680-bib-0036] Prola, A. , Pires da Silva, J. , Guilbert, A. , Lecru, L. , Piquereau, J. , Ribeiro, M. , Mateo, P. , Gressette, M. , Fortin, D. , Boursier, C. , Gallerne, C. , Caillard, A. , Samuel, J. L. , François, H. , Sinclair, D. A. , Eid, P. , Ventura‐Clapier, R. , Garnier, A. , & Lemaire, C. (2017). SIRT1 protects the heart from ER stress‐induced cell death through eIF2alpha deacetylation. Cell Death and Differentiation, 24(2), 343–356. 10.1038/cdd.2016.138 27911441 PMC5299716

[acel13680-bib-0037] Ren, Z. , He, H. , Zuo, Z. , Xu, Z. , Wei, Z. , & Deng, J. (2019). The role of different SIRT1‐mediated signaling pathways in toxic injury. Cellular & Molecular Biology Letters, 24, 36. 10.1186/s11658-019-0158-9 31164908 PMC6543624

[acel13680-bib-0038] Rong, L. , Wu, J. , Wang, W. , Zhao, R. P. , Xu, X. W. , & Hu, D. (2016). Sirt 1 activator attenuates the bleomycin‐induced lung fibrosis in mice via inhibiting epithelial‐to‐mesenchymal transition (EMT). European Review for Medical and Pharmacological Sciences, 20(10), 2144–2150. 27249616

[acel13680-bib-0039] Satoh, A. , Brace, C. S. , Rensing, N. , Cliften, P. , Wozniak, D. F. , Herzog, E. D. , Yamada, K. A. , & Imai, S. (2013). Sirt1 extends life span and delays aging in mice through the regulation of Nk2 homeobox 1 in the DMH and LH. Cell Metabolism, 18(3), 416–430. 10.1016/j.cmet.2013.07.013 24011076 PMC3794712

[acel13680-bib-0040] Schafer, S. , Viswanathan, S. , Widjaja, A. A. , Lim, W. W. , Moreno‐Moral, A. , DeLaughter, D. M. , Ng, B. , Patone, G. , Chow, K. , Khin, E. , Tan, J. , Chothani, S. P. , Ye, L. , Rackham, O. J. L. , Ko, N. S. J. , Sahib, N. E. , Pua, C. J. , Zhen, N. T. G. , Xie, C. , … Cook, S. A. (2017). IL‐11 is a crucial determinant of cardiovascular fibrosis. Nature, 552(7683), 110–115. 10.1038/nature24676 29160304 PMC5807082

[acel13680-bib-0041] Shao, D. , Yao, C. , Kim, M. H. , Fry, J. , Cohen, R. A. , Costello, C. E. , Matsui, R. , Seta, F. , McComb, M. , & Bachschmid, M. M. (2019). Improved mass spectrometry‐based activity assay reveals oxidative and metabolic stress as sirtuin‐1 regulators. Redox Biology, 22, 101150. 10.1016/j.redox.2019.101150 30877853 PMC6423473

[acel13680-bib-0042] Sun, J. , He, X. , Zhu, Y. , Ding, Z. , Dong, H. , Feng, Y. , du, J. , Wang, H. , Wu, X. , Zhang, L. , Yu, X. , Lin, A. , McDonald, T. , Zhao, D. , Wu, H. , Hua, W. K. , Zhang, B. , Feng, L. , Tohyama, K. , … Li, L. (2018). SIRT1 activation disrupts maintenance of myelodysplastic syndrome stem and progenitor cells by restoring TET2 function. Cell Stem Cell, 23(3), 355–369 e359. 10.1016/j.stem.2018.07.018 30146412 PMC6143172

[acel13680-bib-0043] Sun, L. , Fan, M. , Huang, D. , Li, B. , Xu, R. , Gao, F. , & Chen, Y. (2021). Clodronate‐loaded liposomal and fibroblast‐derived exosomal hybrid system for enhanced drug delivery to pulmonary fibrosis. Biomaterials, 271, 120761. 10.1016/j.biomaterials.2021.120761 33774524

[acel13680-bib-0044] Sun, W. , Qiao, W. , Zhou, B. , Hu, Z. , Yan, Q. , Wu, J. , Wang, R. , Zhang, Q. , & Miao, D. (2018). Overexpression of Sirt1 in mesenchymal stem cells protects against bone loss in mice by FOXO3a deacetylation and oxidative stress inhibition. Metabolism, 88, 61–71. 10.1016/j.metabol.2018.06.006 30318050

[acel13680-bib-0045] Vyas, N. , Kurian, S. J. , Bagchi, D. , Manu, M. K. , Saravu, K. , Unnikrishnan, M. K. , Mukhopadhyay, C. , Rao, M. , & Miraj, S. S. (2021). Vitamin D in prevention and treatment of COVID‐19: Current perspective and future prospects. Journal of the American College of Nutrition, 40(7), 632–645. 10.1080/07315724.2020.1806758 32870735

[acel13680-bib-0046] Wang, B. , Liu, T. , Wu, J. C. , Luo, S. Z. , Chen, R. , Lu, L. G. , & Xu, M. Y. (2018). STAT3 aggravates TGF‐beta1‐induced hepatic epithelial‐to‐mesenchymal transition and migration. Biomedicine & Pharmacotherapy, 98, 214–221. 10.1016/j.biopha.2017.12.035 29268242

[acel13680-bib-0047] Wei, J. , Ghosh, A. K. , Chu, H. , Fang, F. , Hinchcliff, M. E. , Wang, J. , Marangoni, R. G. , & Varga, J. (2015). The histone deacetylase Sirtuin 1 is reduced in systemic sclerosis and abrogates fibrotic responses by targeting transforming growth factor beta signaling. Arthritis & Rhematology, 67(5), 1323–1334. 10.1002/art.39061 PMC4518870 25707573

[acel13680-bib-0048] Wu, K. , Li, B. , Lin, Q. , Xu, W. , Zuo, W. , Li, J. , Liu, Q. , Tu, T. , Zhang, B. , Xiao, Y. , & Liu, Q. (2021). Nicotinamide mononucleotide attenuates isoproterenol‐induced cardiac fibrosis by regulating oxidative stress and Smad3 acetylation. Life Sciences, 274, 119299. 10.1016/j.lfs.2021.119299 33675899

[acel13680-bib-0049] Xiong, J. , Zhuang, T. , Ma, Y. , Xu, J. , Ye, J. , Ma, R. , Zhang, S. , Liu, X. , Liu, B. F. , Hao, C. , Zhang, G. , & Chen, Y. (2021). Optimization of bifunctional piperidinamide derivatives as sigma1R antagonists/MOR agonists for treating neuropathic pain. European Journal of Medicinal Chemistry, 226, 113879. 10.1016/j.ejmech.2021.113879 34628236

[acel13680-bib-0050] Xu, C. , Wang, L. , Fozouni, P. , Evjen, G. , Chandra, V. , Jiang, J. , Lu, C. , Nicastri, M. , Bretz, C. , Winkler, J. D. , Amaravadi, R. , Garcia, B. A. , Adams, P. D. , Ott, M. , Tong, W. , Johansen, T. , Dou, Z. , & Berger, S. L. (2020). SIRT1 is downregulated by autophagy in senescence and ageing. Nature Cell Biology, 22(10), 1170–1179. 10.1038/s41556-020-00579-5 32989246 PMC7805578

[acel13680-bib-0051] Yang, R. , Zhang, J. , Li, J. , Qin, R. , Chen, J. , Wang, R. , Goltzman, D. , & Miao, D. (2022). Inhibition of Nrf2 degradation alleviates age‐related osteoporosis induced by 1,25‐Dihydroxyvitamin D deficiency. Free Radical Biology & Medicine, 178, 246–261. 10.1016/j.freeradbiomed.2021.12.010 34890768

[acel13680-bib-0052] Zeng, Z. , Cheng, S. , Chen, H. , Li, Q. , Hu, Y. , Wang, Q. , Zhu, X. , & Wang, J. (2017). Activation and overexpression of Sirt1 attenuates lung fibrosis via P300. Biochemical and Biophysical Research Communications, 486(4), 1021–1026. 10.1016/j.bbrc.2017.03.155 28365154

[acel13680-bib-0053] Zerr, P. , Palumbo‐Zerr, K. , Huang, J. , Tomcik, M. , Sumova, B. , Distler, O. , Schett, G. , & Distler, J. H. (2016). Sirt1 regulates canonical TGF‐beta signalling to control fibroblast activation and tissue fibrosis. Annals of the Rheumatic Diseases, 75(1), 226–233. 10.1136/annrheumdis-2014-205740 25180292

[acel13680-bib-0054] Zhang, Y. , Fang, F. , Tang, J. , Jia, L. , Feng, Y. , Xu, P. , & Faramand, A. (2019). Association between vitamin D supplementation and mortality: Systematic review and meta‐analysis. British Medical Journal, 366, l4673. 10.1136/bmj.l4673 31405892 PMC6689821

[acel13680-bib-0055] Zhao, H. , Wang, Z. , Tang, F. , Zhao, Y. , Feng, D. , Li, Y. , Hu, Y. , Wang, C. , Zhou, J. , Tian, X. , & Yao, J. (2018). Carnosol‐mediated Sirtuin 1 activation inhibits enhancer of Zeste homolog 2 to attenuate liver fibrosis. Pharmacological Research, 128, 327–337. 10.1016/j.phrs.2017.10.013 29106960

[acel13680-bib-0056] Zisman, D. A. , Keane, M. P. , Belperio, J. A. , Strieter, R. M. , & Lynch, J. P., 3rd. (2005). Pulmonary fibrosis. Methods in Molecular Medicine, 117, 3–44. 10.1385/1-59259-940-0:003 16130230 PMC7120641

