# Supplementary material for: Sirt1 overexpression improves senescence‐associated pulmonary fibrosis induced by vitamin D deficiency through downregulating IL‐11 transcription
Source: Aging Cell. 2022 Jul 30;21(8):e13680. doi: 10.1111/acel.13680 (PMC9381906; doi:10.1111/acel.13680)
Supplement: Supplementary file 1 — Appendix S1. [file ACEL-21-e13680-s001.zip › revised acel13680-sup-0001-Supinfo/ACEL_13680_SI2_Figures S1-S4 Legends.docx]

**SI2 Figure S1-S4 Legends**

**Figure S1 The pulmonary p16- and p53-positive cells and serum IL-11 level increased, however, serum 1,25(OH)_2_D_3_ level decreased in physiologically aged mice compared with young mice**

(A) Representative micrographs of immunochemical or immunohistochemical showed pulmonary alveoli and bronchus staining for p16 and p53. (B) Alveolar positive cells or area for p16 and p53. (C) Bronchial positive cells or area for p16 and p53. (D-E) Serum 1,25(OH)_2_D_3_ and IL-11 levels detected with ELISA assay. Values are the mean ± SEM of three determinations per group. *P < 0.05, **P < 0.01, ***P < 0.001 compared with the young mice.

**Figure S2 Sirt1 overexpression improves pulmonary aging in VD deficiency mice**

(A) Representative micrographs of immunochemical or immunohistochemical showed pulmonary alveoli and bronchus staining for senescence associated-β-galactosidase (SA-β-gal), p16 and p53. (B) Percentage of cells positive for SA-β-gal. (C) Alveolar positive cells or area for p16 and p53. (D) Bronchial positive cells or area for p16 and p53. (E) Western blotting of pulmonary extracts showing p16, p19, p21 and p53. β-actin was used as the loading control. (F) Protein levels relative to β-actin were assessed by densitometric analysis. Six mice per group were used for experiments. Values are the mean ± SEM from six determinations per group. *P < 0.05, **P < 0.01, ***P < 0.001 compared with the WT group; ^###^P < 0.001 compared with the *Sirt1^Tg^* group; ^&^P < 0.05, ^&&^P < 0.01, ^&&&^P < 0.001 compared with the *Cyp27b1*^−^*^/^*^−^ group.

**Figure S3 Sirt1 overexpression improves pulmonary DNA damage and SASP in VD deficiency mice**

(A) Representative micrographs of immunochemical or immunohistochemical showed pulmonary alveoli and bronchus staining for 8-hydroxyguanosine (8-OHdG), CD3e, IL-1β, IL-6, and TNF-α. (B) Alveolar positive cells or area for 8-OHdG, CD3e, IL-1β, IL-6, and TNF-α. (C) Bronchial positive cells or area for 8-OHdG, CD3e, IL-1β, IL-6, and TNF-α. (D) Western blotting of pulmonary extracts showing Chk2, p-Chk2(Thr68), γ-H2A.X(Ser139), p65, p-p65(Ser536), IkB-α, p-IkB-α(Ser32), IL-1β and IL-6. β-actin was used as the loading control. (E) Protein levels relative to β-actin were assessed by densitometric analysis. Six mice per group were used for experiments. Values are the mean ± SEM from six determinations per group. *P < 0.05, **P < 0.01, ***P < 0.001 compared with the WT group; ^#^P < 0.05, ^##^P < 0.01, ^###^P < 0.001 compared with the *Sirt1^Tg^* group; ^&^P < 0.05, ^&&^P < 0.01, ^&&&^P < 0.001 compared with the *Cyp27b1*^−^*^/^*^−^ group.

**Figure S4 Sirt1 negatively regulates IL-11 expression through deacetylating H3K9/14ac in *IL-11* promoter, mainly the binding region of Smad2**

Pulmonary fibroblasts were isolated from 9-week-old WT mice and treated by TGF-β1 (5 ng mL^−1^ 48 h), and SRT1720 (2.5 μM 48 h) or Ex527 (10 μM 48 h). (A) Western blotting for Sirt1, H3K9/14ac, Smad2, p-Smad2(Ser465/467), p-Smad2/3(Ser423/425) and IL-11. β-actin was used as the loading control. (B) Protein levels relative to β-actin were assessed by densitometric analysis. Three biological replicates were used per experiment. Values are means ± SEM of six determinations. **P < 0.01, ***P < 0.001 compared with control group; ^#^P < 0.05, ^##^P < 0.01, ^###^P < 0.001 compared with TGF-β1-treated group; ^&&^P < 0.01, ^&&&^P < 0.001 compared with TGF-β1- and SRT1720-treated group. Human embryonic lung fibroblasts MRC-5 were treated by TGF-β1 (5 ng mL^−1^ 48 h), and SRT1720 (2.5 μM 48 h) or Ex527 (10 μM 48 h). (C) ChIP assays were performed with chromatin prepared from MRC-5 cells treated with TGF-β1 and SRT1720 or Ex527. The chromatin was immunoprecipitated with normal rabbit IgG or antibodies against Smad2, and precipitated genomic DNA was analyzed as fold enrichment through real-time PCR using different primers for the different regions of *IL-11* promoter. The β-actin promoter (−204 to −59 bp) was used as a negative control. Three biological replicates were used per experiment. Values are means ± SEM of six determinations. *P < 0.05, **P < 0.01, ***P < 0.001 compared with control group; ^#^P < 0.05, ^##^P < 0.01 compared with TGF-β1-treated group; ^&^P < 0.05, ^&&^P < 0.01, ^&&&^P < 0.001 compared with TGF-β1- and SRT1720- treated group.
