# Supplementary material for: Sirt1 overexpression improves senescence‐associated pulmonary fibrosis induced by vitamin D deficiency through downregulating IL‐11 transcription
Source: Aging Cell. 2022 Jul 30;21(8):e13680. doi: 10.1111/acel.13680 (PMC9381906; doi:10.1111/acel.13680)
Supplement: Supplementary file 1 — Appendix S1. [file ACEL-21-e13680-s001.zip › revised acel13680-sup-0001-Supinfo/ACEL_13680_SI3_Table S1.docx]

**SI3 Table S1**

**Primers for Real Time RT-PCR**

| Name | S/AS | Sequence | Primer (bp) | Species | Tm  (°C) | Length  (bp) |
| --- | --- | --- | --- | --- | --- | --- |
| *Human-IL-11*  (-187~-112) | S | 5’-CAACTTTTCCTTCCGTGCCC-3’ | 20 | human | 55 | 76 |
|  | AS | 5’-TTTCCATTCAGACGGAGCGG-3’ | 20 |  |  |  |
| *Human-IL-11*  (-577~-362) | S | 5’-CCCCTCTGATCCTCTTTGCT-3’ | 20 | human | 56 | 216 |
|  | AS | 5’-AGGACGGAAAGGCAGAGAAA-3’ | 20 |  |  |  |
| *Human-IL-11*  (-871~-724) | S | 5’-GGCTTTTACTCTCTGCCCCC-3’ | 20 | human | 55 | 148 |
|  | AS | 5’-TGAAGAAAGGCACCAGGCAA-3’ | 20 |  |  |  |
| *Human-IL-11*  (-1275~-1054) | S | 5’-TGAGCTGAGATCACACCACT-3’ | 20 | human | 56 | 221 |
|  | AS | 5’-CCACCACACCCGGCTAATTT-3’ | 20 |  |  |  |
| *Human-IL-11*  (-1535~-1252) | S | 5’-CTCCACCTGCCTCTTTGAGA-3’ | 20 | human | 55 | 283 |
|  | AS | 5’-GTGCAGTGGTGTGATCTCAG-3’ | 20 |  |  |  |
| *Human-IL-11*  (-1817~-1736) | S | 5’-CCATCTCTCTCGAAGGCGAC-3’ | 21 | human | 54 | 81 |
|  | AS | 5’-TGACCATCCTTATTCCCCTCG-3’ | 21 |  |  |  |
| *Human-IL-11*  (-1816~-1739) | S | 5’-CATCTCTCTCGAAGGCGACA-3’ | 20 | human | 54 | 78 |
|  | AS | 5’-ACCATCCTTATTCCCCTCGTTA-3’ | 22 |  |  |  |
| *Human-IL-11*  (-1843~-1771) | S | 5’-CGTCTTCACCCCATCTTGCT-3’ | 20 | human | 55 | 73 |
|  | AS | 5’-ACACATCAGGGACACAGAGA-3’ | 20 |  |  |  |
| *Human-IL-11*  (-1994~-1824) | S | 5’-CCACTGTGAGCCTGGGTAG-3’ | 19 | human | 56 | 171 |
|  | AS | 5’-AGCAAGATGGGGTGAAGACG-3’ | 20 |  |  |  |
| *Human-β-actin promoter* (-204~-59) | S | 5’-TCCTCCTCTTCCTCAATCTCG-3’ | 21 | human | 56 | 146 |
|  | AS | 5’-AAGGCAACTTTCGGAACGG-3’ | 19 |  |  |  |
| *IL-11* | S | 5’-ATGAACTGTGTTTGCCGCCT-3’ | 20 | human | 60 | 200 |
|  | AS | 5’-TCAGCTGGGAATTTGTCCCTC-3’ | 21 |  |  |  |
| *Smad2* | S | 5’-CGTCCATCTTGCCATTCACG-3’ | 20 | human | 60 | 182 |
|  | AS | 5’-CTCAAGCTCATCTAATCGTCCTG-3’ | 23 |  |  |  |
| *Gapdh* | S | 5’-GGAGCGAGATCCCTCCAAAAT-3’ | 21 | human | 60 | 197 |
|  | AS | 5’-GGCTGTTGTCATACTTCTCATGG-3’ | 23 |  |  |  |
| *p16* | S | 5’-CCCGATTCAGGTGATGATGAT-3’ | 21 | mouse | 55 | 100 |
|  | AS | 5’-GCGGGAGAAGGTAGTGG-3’ | 17 |  |  |  |
| *Tgfb1* | S | 5’-TAAAATCAAGTGTGGAGCAAC-3’ | 21 | mouse | 55 | 119 |
|  | AS | 5’-GTCAAAAGACAGCCACTCAG-3’ | 20 |  |  |  |
| *IL-11* | S | 5’-TGTTCTCCTAACCCGATCCCT-3’ | 21 | mouse | 60 | 149 |
|  | AS | 5’-CAGGAAGCTGCAAAGATCCCA-3’ | 21 |  |  |  |
| *acta2* | S | 5’-GTCCCAGACATCAGGGAGTAA-3’ | 21 | mouse | 60 | 103 |
|  | AS | 5’-TCGGATACTTCAGCGTCAGGA-3’ | 21 |  |  |  |
| *β-actin* | S | 5’-GGCTGTATTCCCCTCCATCG-3’ | 20 | mouse | 60 | 154 |
|  | AS | 5’-CCAGTTGGTAACAATGCCATGT-3’ | 22 |  |  |  |

S, sense; AS, antisense, sequence; Tm, annealing temperature; length, amplicon
