# Supplementary material for: Sirt1 overexpression improves senescence‐associated pulmonary fibrosis induced by vitamin D deficiency through downregulating IL‐11 transcription
Source: Aging Cell. 2022 Jul 30;21(8):e13680. doi: 10.1111/acel.13680 (PMC9381906; doi:10.1111/acel.13680)
Supplement: Supplementary file 1 — Appendix S1. [file ACEL-21-e13680-s001.zip › revised acel13680-sup-0001-Supinfo/ACEL_13680_SI4_Predicting the binding sites of Smad2 or Smad3 in IL-11 promoter.docx]

**SI4 Predicting the binding sites of Smad2 or Smad3 in *IL-11* promoter**

1. Smad2


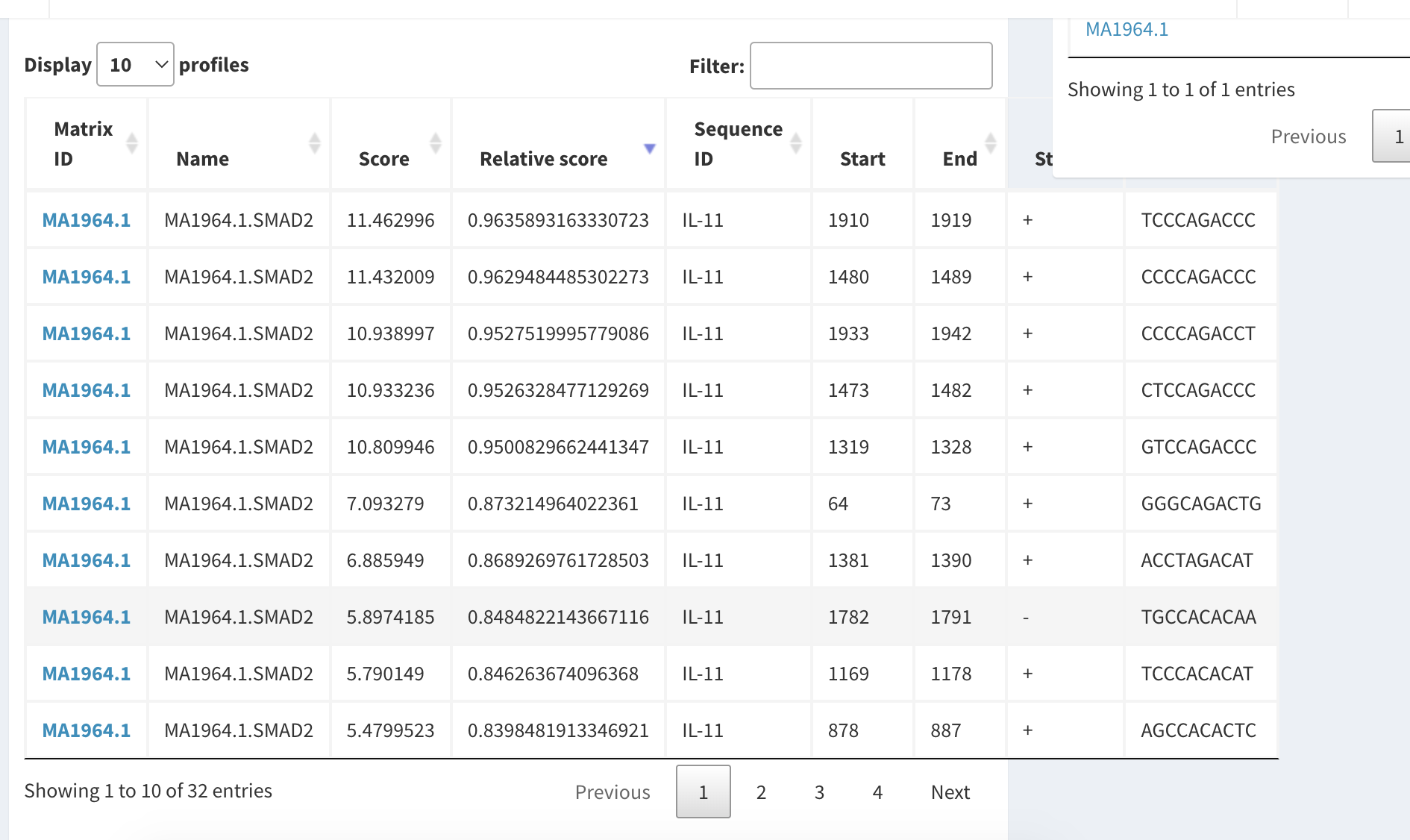


1. Smad3


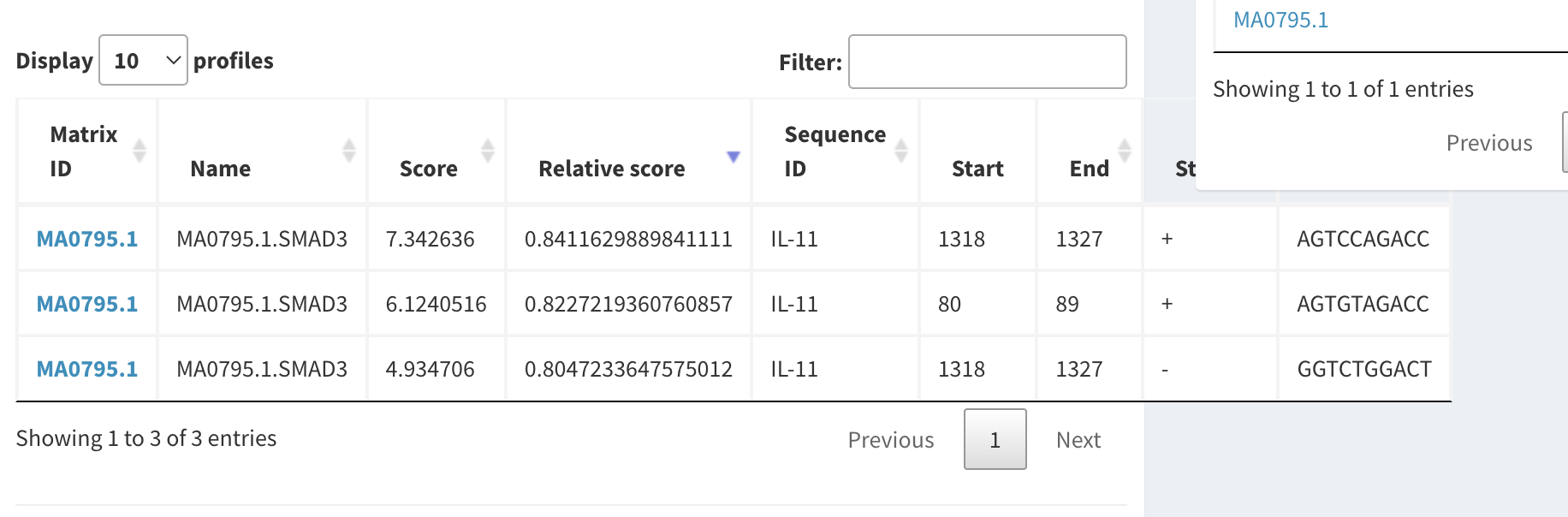


When we used the website (https://jaspar.genereg.net/) to predict the binding sites of Smad2 or Smad3 in *IL-11* promoter, we found that Smad2 had more binding sites than Smad3. Smad2 had 32 predicted binding sites, while Smad3 only had 3 binding sites.
