# Supplementary figures and images for: Sirt1 overexpression improves senescence‐associated pulmonary fibrosis induced by vitamin D deficiency through downregulating IL‐11 transcription
Source: Aging Cell. 2022 Jul 30;21(8):e13680. doi: 10.1111/acel.13680 (PMC9381906; doi:10.1111/acel.13680)

Figure S1

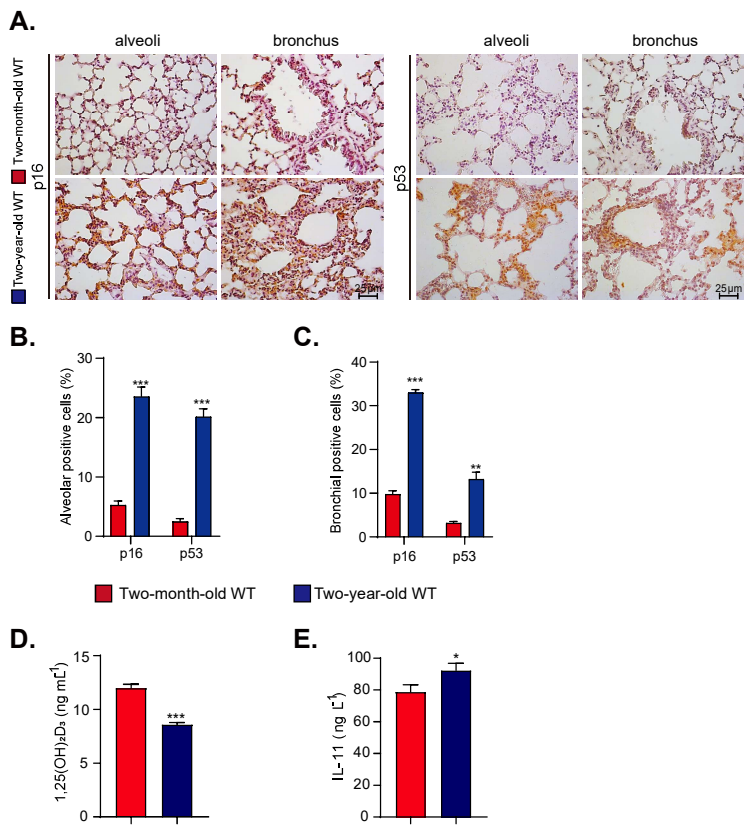

**Figure S2**

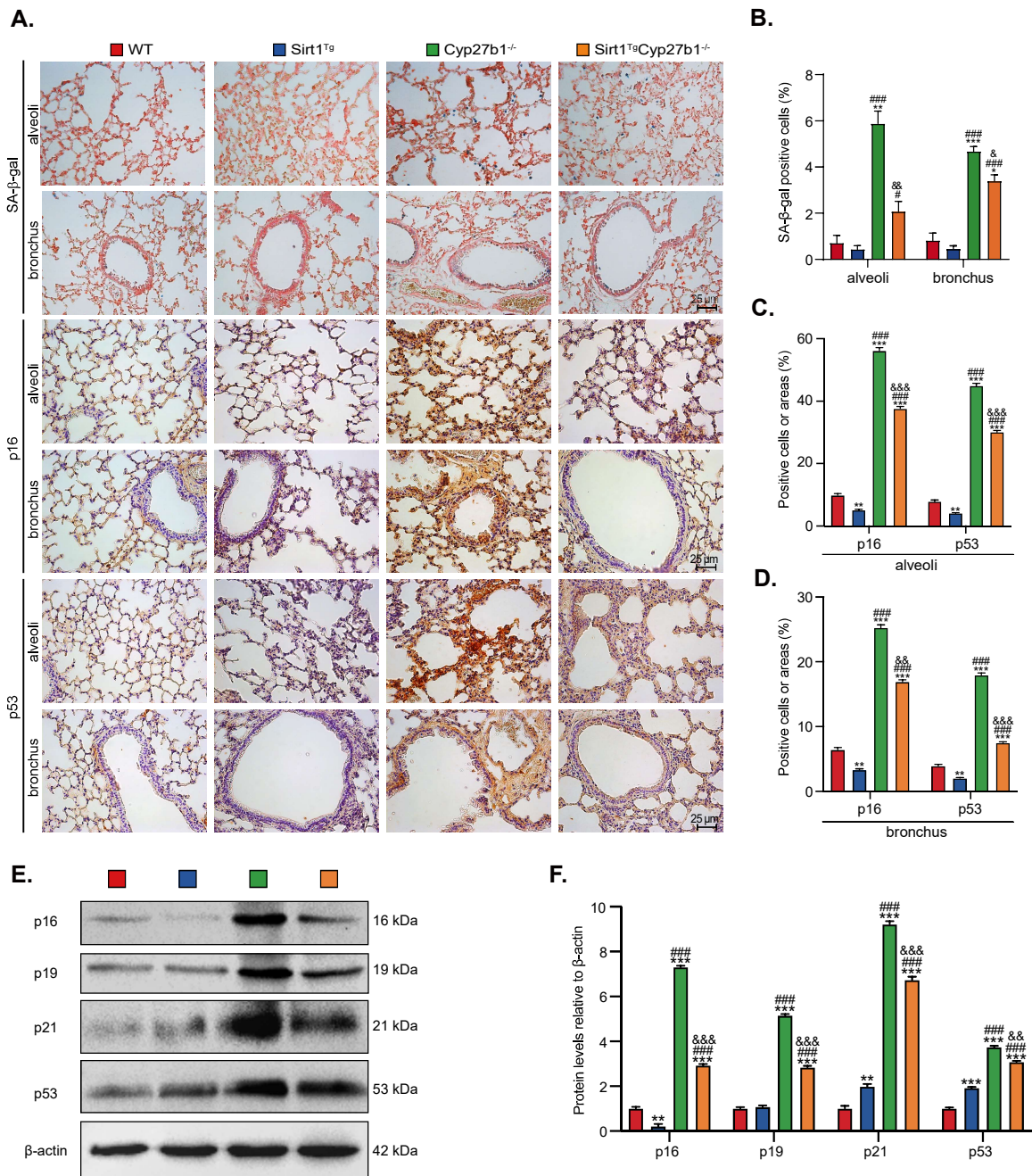

Figure S3

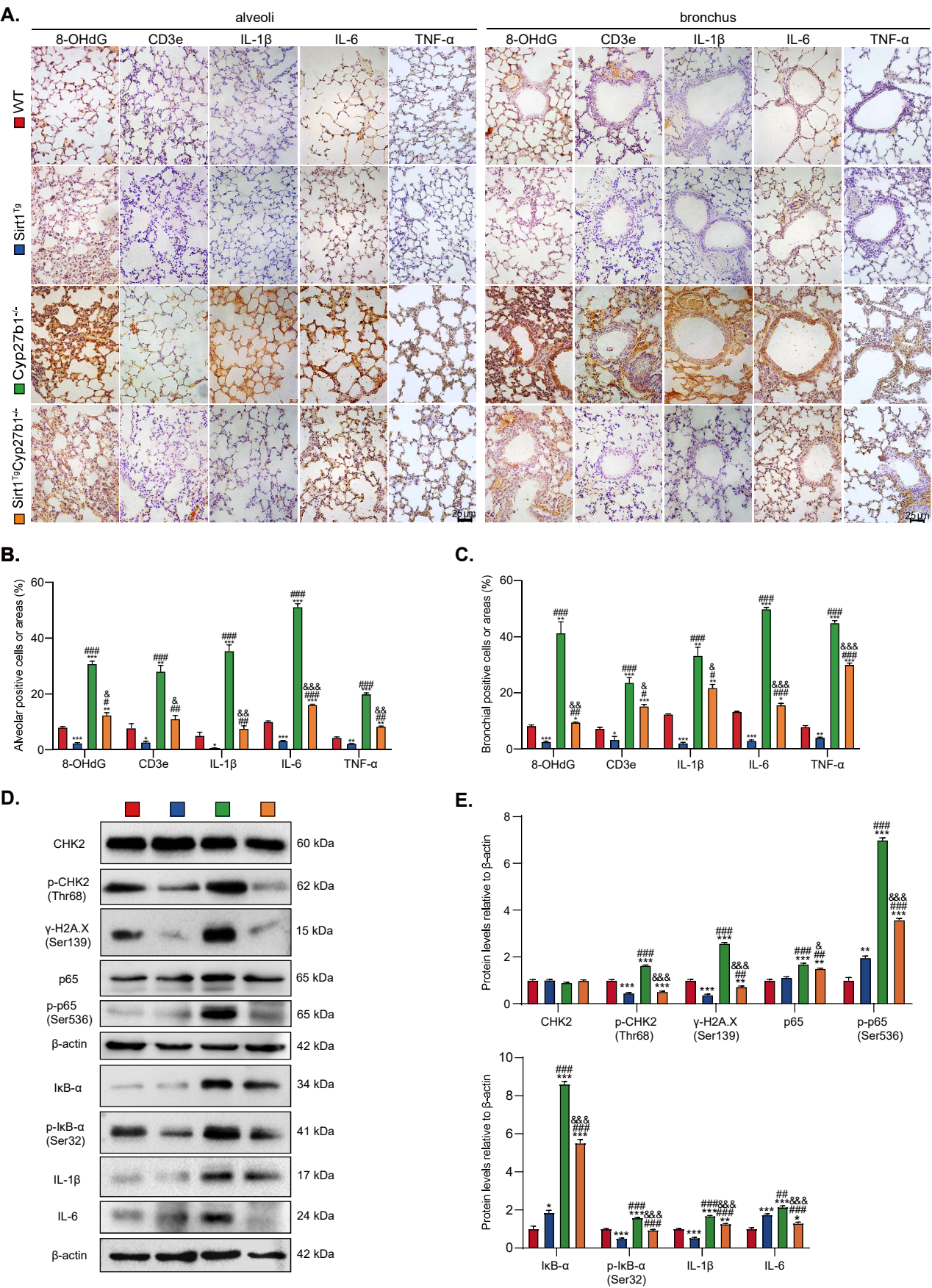

Figure S4

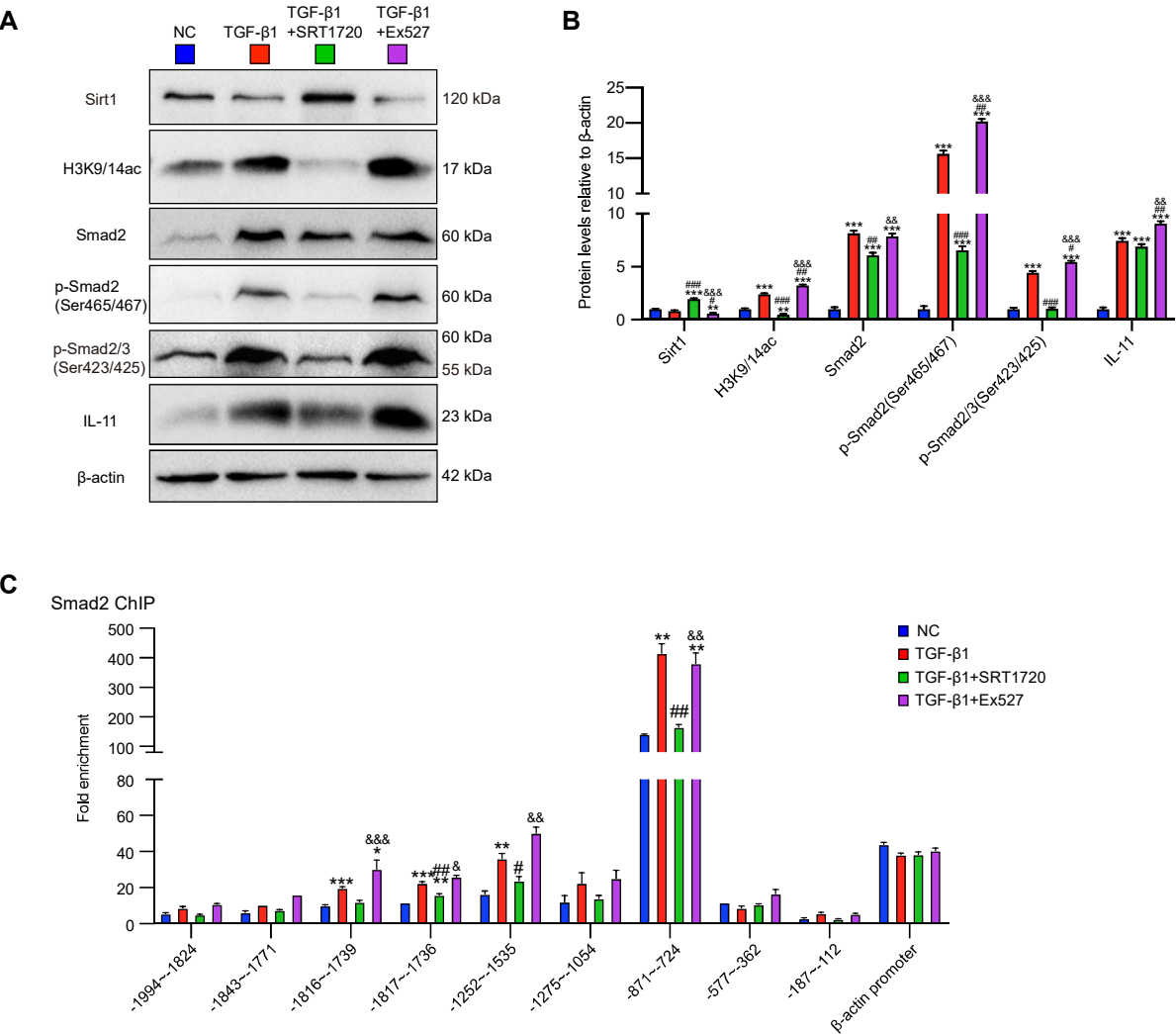

Supplement: Supplementary file 1 — Appendix S1. [file ACEL-21-e13680-s001.zip › revised acel13680-sup-0001-Supinfo/ACEL_13680_SI1_Figures S1-S4.pdf]
